# Radiation-induced cellular plasticity primes glioblastoma for forskolin-mediated differentiation

**DOI:** 10.1073/pnas.2415557122

**Published:** 2025-02-26

**Authors:** Ling He, Daria Azizad, Kruttika Bhat, Angeliki Ioannidis, Carter J. Hoffmann, Evelyn Arambula, Mansoureh Eghbali, Aparna Bhaduri, Harley I. Kornblum, Frank Pajonk

**Affiliations:** ^a^Department of Radiation Oncology, David Geffen School of Medicine at University of California, Los Angeles, CA 90095; ^b^Jonsson Comprehensive Cancer Center at University of California, Los Angeles, CA 90095; ^c^Department of Biological Chemistry at University of California, Los Angeles, CA 90095; ^d^Department of Anesthesiology at University of California, Los Angeles, CA 90095; ^e^Neuropsychiatric Institute-Semel Institute for Neuroscience and Human Behavior at University of California, Los Angeles, CA 90095; ^f^Department of Neurosurgery, David Geffen School of Medicine at University of California, Los Angeles, CA 90095

**Keywords:** glioblastoma, glioma stem cells, radiotherapy

## Abstract

Glioblastoma (GBM) is the deadliest brain cancer in adults, and all patients succumb to the tumor. While surgery followed by chemoradiotherapy delays disease progression, these treatments do not lead to tumor control, and targeted therapies or biologics have failed to further improve survival. Utilizing a transient radiation-induced state of multipotency, we used the adenylcyclase activator forskolin to alter the fate of irradiated glioma cells. The effects of the combined treatment on neuronal marker expression, cell cycle distribution, and proliferation were studied. Gene expression profiling was conducted using bulk RNA-seq. Changes in cell populations were investigated using single-cell RNA-seq. Effects on glioma stem cells (GSCs) were studied in extreme limiting dilution assays, and the effects on median survival were studied in both syngeneic and PDOX mouse models of GBM. The combined treatment induced the expression of neuronal markers in glioma cells, reduced proliferation, and led to a distinct gene expression profile. scRNA-seq revealed that the combined treatment forced glioma cells into a microglia- and neuron-like phenotype. In vivo, this treatment led to a loss of GSCs and prolonged median survival. Collectively, our data suggest that revisiting a differentiation therapy with forskolin in combination with radiation could lead to clinical benefit.

## Significance

The distinctive nature of glioblastoma (GBM) and the blood–brain barrier (BBB) are obstacles for therapies that aim to permanently stop glioma cells from dividing. Similar to reprogramming normal cells, “differentiation therapies” try to push cancer cells into a flexible state and then guide them to differentiate into nondividing cells. Our study shows that using radiation first and then activating adenylate cyclase can solve the problems found in earlier work. Irradiation induces a flexible cellular state, and the addition of an adenylate cyclase activator helps turn them into types that cannot regrow tumors. This combined approach works well against GBM and suggests that using radiotherapy together with carefully timed differentiation treatments that force cells to mature could improve treatment outcome.

Glioblastoma (GBM) is the deadliest brain tumor in adults. The current standard of care, surgery followed by chemoradiotherapy, has not changed in almost two decades, and the median survival times of 15 to 18 mo are unacceptably low. A large body of literature supports the hierarchical organization of GBM with glioma stem cells (GSCs) at the top of this hierarchy, able to regrow the tumor and to give rise to more differentiated GBM cells (1, 2). Importantly, GSCs resist established anticancer therapies, making them a main culprit in treatment failure (3, 4).

The debate over the identity of the cell of origin for GBM is not settled, but neural stem cells (NSCs) and oligodendrocyte precursor cells (OPCs) are likely candidates (5), and GSCs share stem cell traits with these cell populations. While the stem cell/precursor state of NSCs and OPCs depends on the association with a supporting niche environment, GSCs utilize the same niche factors but also carry a mutational burden that supports stemness (6) and actively modulate prodifferentiation signaling to maintain GSCs traits and to block differentiation (7). Overcoming such differentiation blocks in solid cancers has been attempted in the past. While numerous approaches have been successful in vitro (8–13), few succeeded in vivo (11, 12) and even fewer prolonged survival (12). In GBM, this situation is further complicated by the requirement for systemically administered drug-based differentiation therapies to cross the blood–brain barrier (BBB).

We recently reported that ionizing radiation—aside from inducing cell death—led to global epigenetic changes in surviving cells with a transient acquisition of an open chromatin state in the promoter region of developmental transcription factors (14, 15). Subsequently, some surviving nonstem cancer cells converted their phenotype into induced cancer stem cells (16, 17), and some surviving GSCs transdifferentiated into pericyte- and vascular-like cells (18), thus suggesting that exposure to ionizing radiation transiently elevates glioma cells into a multipotent state, comparable to lineage-committed normal stem/progenitor cells.

Ideally, a differentiation therapy would direct cells toward a mitotically incompetent state. E.g., sarcoma cells can be forced into erythrocyte-like cells in vitro that even expel their nucleus (19). In the central nervous system (CNS), neurons are terminally differentiated cells that have lost their capacity to divide, thus making them a desirable end-state for a differentiation therapy in GBM. In the present study, we sought to use this effect of ionizing radiation in combination with forskolin, an established agent for neuronal differentiation, to drive glioma cells into terminal differentiation.

## Results

### Treatment of Irradiated Glioma Cells with dbcAMP Leads to Expression of Neuronal Markers.

**Fig. 1.**
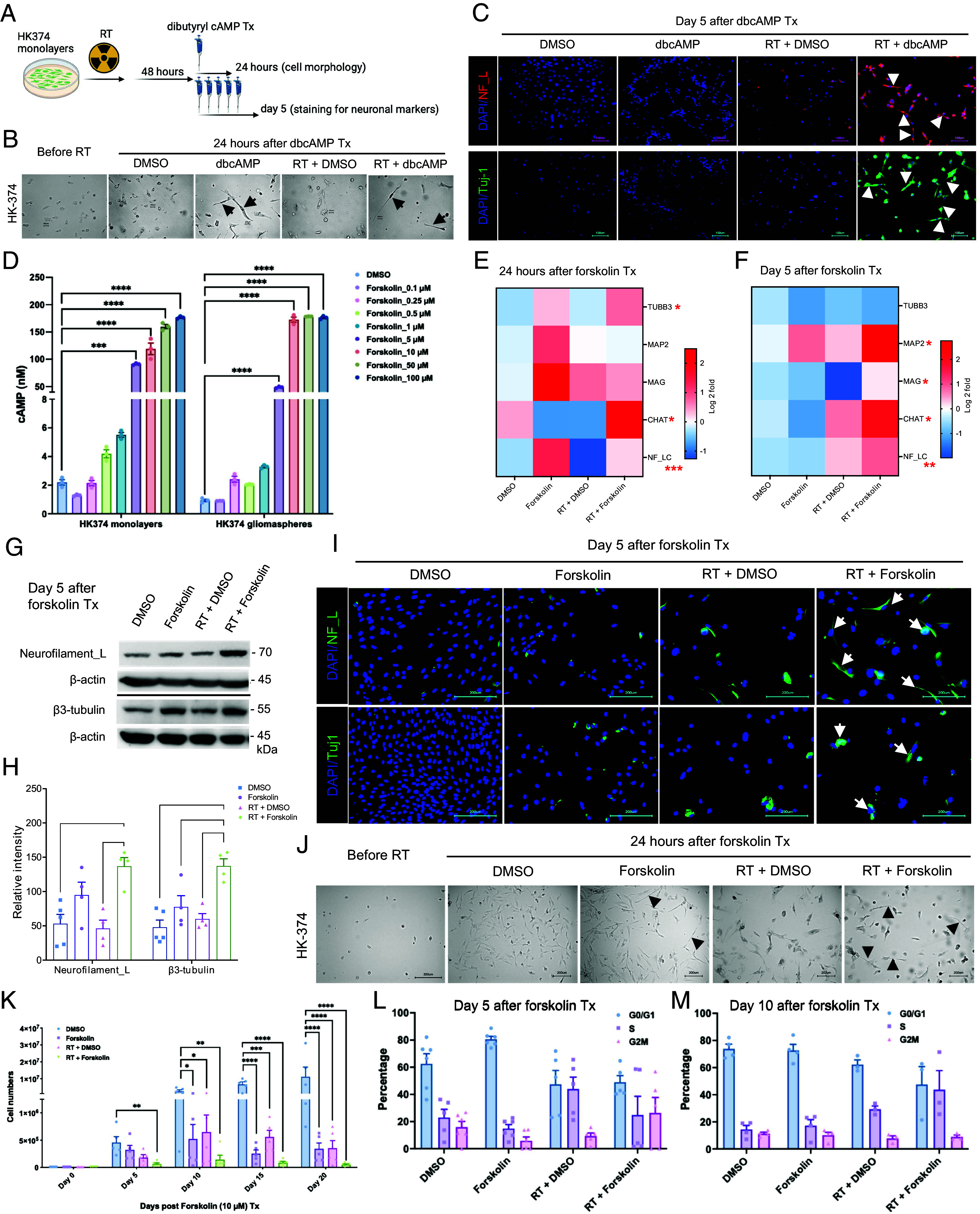
Neuronal marker induction by dbcAMP or forskolin. Schematic of the experimental design for Fig. 1 (*A*). Cells showed morphology changes into neuron-like cells (black arrows) as early as 24 h after dbcAMP treatment start (*B*). The combination of radiation and dbcAMP but not radiation or dbcAMP treatment alone induced expression of neurofilament light chain (NF_L) and Tuj-1 (white arrows) (*C*). Treatment of HK-374 glioma monolayer cells and glioma spheres with forskolin leads to a dose-dependent induction of intracellular cAMP levels (*D*) and expression of neuronal markers at 24 h (*E*) and 5 d after start of forskolin treatment (*F*). Representative western blot for NF_L and β3-tubulin (*G*) shows a significant induction of both proteins after treatment with radiation and forskolin (*H*). Immunofluorescent imaging of glioma cells treated with radiation and forskolin shows expression of NF_L and Tuj-1 in a subset of cells with elongated cell bodies (white arrows) (*I*). Morphology changes after irradiation and forskolin treatment could be observed as early as 24 h after start of forskolin treatment (*J*, black arrows). Treatment of HK-374 glioma cells with radiation and consecutive treatment with forskolin for 5, 10, 15, or 20 d significantly inhibits cell proliferation (*K*) and alters the cell cycle distribution (L, day 5; M, day 10). N = 3 to 6, where N represents the number of independent biological samples conducted). *P-*values were calculated using one-way ANOVA for *D*, *H*, and *K*; Two-way ANOVA for *L* and *M*; The *P*-values listed in the heatmaps were from the comparison of RT + Forskolin to RT + DMSO. *P-value < 0.05, **P-value < 0.01, ***P-value < 0.001, and ****P-value < 0.0001.

In a first set of experiments, we tested the hypothesis that radiation induced a state multipotency that could be used to direct glioma cells into a neuron-like state using dbcAMP, part of established differentiation protocols for the neuronal differentiation of induced pluripotent stem cells (iPS cells) (20, 21). Patient-derived HK-374 glioma cells were irradiated with 0 or 4 Gy. We had previously shown that 48 h after a single dose of 4 Gy, epigenetic remodeling led to a multipotent state with gains in open chromatin in the promoter regions of developmental transcription factors (15). Therefore, we treated irradiated and unirradiated cells with dbcAMP (daily, 1 mM), starting 48 h after irradiation (Fig. 1*A*). As early as 24 h after start of treatment, both dbcAMP-treated unirradiated and irradiated cells showed elongated cell bodies reminiscent of neuron-like morphology (Fig. 1*B*, black arrows; *SI Appendix,* Fig. S1*A*) while control cells, not treated with dbcAMP, retained the morphology of untreated cells (Fig. 1*B*).

After five daily treatments with dbcAMP, irradiated cells showed strong expression of the neuronal markers Tuj-1 and Neurofilament Light Chain (NF-LC) (Fig. 1*C*, white arrows), which agreed with the neuron-like morphology of these cells. Cells treated with radiation or dbcAMP alone only showed faint expression of both markers (Fig. 1*C*).

### The Adenylate Cyclase Activator Forskolin Induces Neuron-Like Phenotype in Irradiated Glioma Cells.

In vivo, direct application of dbcAMP is not a feasible approach to differentiate glioma cells. To overcome this limitation, we next tested whether forskolin, a known activator of adenylate cyclase (22) could increase cAMP levels in glioma cells. Glioma cells grown as monolayers and gliomaspheres enriched for GSCs were treated with forskolin at concentrations of 0.1, 0.25, 0.5, 1, 5, 10, 50, or 100 µM. Differentiated cells and cells enriched for GSCs both showed a dose-dependent increase in cAMP production, thus confirming that forskolin can induce cAMP production in glioma cells, including GSCs. Induced intracellular cAMP concentrations did not reach mM concentrations used in dbcAMP experiments but plateaued below 200 nM after 15 min of forskolin treatment (Fig. 1*D*).

Next, we tested whether treatment of irradiated glioma cells with forskolin would also induce the expression of neuronal markers. Quantitative RT-PCR 24 h and 5 d after the start of forskolin treatment revealed that combined treatment with radiation and forskolin increased the expression of the neuronal markers β3-tubulin, CHAT, NF-LC, and MAG, thus indicating possible induction of a differentiated cell state (Fig. 1 *E* and *F*). We further confirmed the induction of neuronal markers NF-LC and β3-tubulin on day 5 after irradiation and forskolin treatment by western blotting (Fig. 1 *G* and *H*) and immunofluorescence staining (Fig. 1*I*, white arrows). However, expression of these markers was less uniform than after treatment with dbcAMP, consistent with much lower intracellular cAMP concentrations (Fig. 1*D*). Forskolin alone or in combination with radiation-induced changes in cell morphology with elongation of some of the cells’ bodies (Fig. 1*J*, black arrows; *SI Appendix,* Fig. S1*B*). Considering the distinct characteristics of different TCGA subtypes in GBM, we included another two patient-derived GBM lines (*SI Appendix,* Table S2) to assess the effect of radiation plus forskolin on neuron-like differentiation. In HK-308 cells (mesenchymal subtype) combined treatment with radiation and forskolin showed similar induction of neuronal markers (*SI Appendix,* Fig. S1*C*), while these effects were less notable in HK-157 cells (proneural subtype) (*SI Appendix,* Fig. S1*D*).

### Forskolin in Combination with Radiation Impacts Cell Proliferation and Cell Cycle in Glioma Cells.

True differentiation toward a neuronal phenotype would result in a loss of mitotic capacity. To test the proliferative capacity of cells treated with radiation and/or forskolin, HK-374, HK-308, or HK-157 patient-derived glioma cells were seeded and treated with 0 or 4 Gy of radiation the following day. After an additional 48 h, cells were treated with DMSO or forskolin (daily at 10 µM). Cell numbers were counted on days 5, 10, 15, and 20 after forskolin treatment initiation. While radiation or forskolin alone reduced cell numbers and/or slowed proliferation in all three lines to some extent, the combination of radiation and forskolin had additive effects on cell numbers from day 5 on (Fig. 1*K* and *SI Appendix,* Fig. S1 *E* and *F*), thus suggesting that the combined effect is not cell line specific. Forskolin is known to affect cell cycle progression through specific inhibition of G1-to-S phase progression (23). To study the lack of proliferation in more detail, we next performed a cell cycle analysis. On day 5, forskolin treatment led to an arrest in the G_1_/G_0_ phase of the cell cycle. Irradiation with a single dose of 4 Gy decreased the number of cells in the G_1_/G_0_- and G_2_/M-phase and increased the population of cells in the S-phase, consistent with the well-known initial arrest at the G_1_/G_0_ and G_2_/M checkpoints and the subsequent release after DNA repair. Combined treatment with radiation and forskolin decreased the number of cells in G_1_/G_0_ while elevating the size of the S- and G_2_/M-phase cell populations (Fig. 1*L*).

On day 10, the G_1_/G_0_ population of untreated control cells increased, consistent with growth inhibition in confluent monolayer cultures. The cell cycle distribution of forskolin-treated cells was similar to the distribution on day 5, while irradiated cells continued to redistribute, matching distributions of untreated control cells. Irradiated cells treated with forskolin appeared to be arrested at the G_1_-to-S transition of the cell cycle (Fig. 1*M*).

Next, we assessed whether the combined treatment would lead to increased numbers of residual DNA double-strand breaks. Using γ-H2AX staining 24 h after forskolin treatment (72 h postirradiation) we found that the addition of forskolin significantly reduced the number of residual γ-H2AX foci, thus excluding enhanced DNA damage as an explanation for the lack of proliferating cells (*SI Appendix,* Fig. S2 *A*–*C*).

To test whether the reduction in proliferation after combined treatment was due to an induction of a quiescent state, we measured RNA and DNA content of the cells. At day 5, combined treatment significantly reduced the number of cells in the G_0_ phase of the cell cycle (*SI Appendix,* Fig. S2 *D* and *E*).

Next, we tested whether forskolin treatment would affect autophagy. Western blotting against LC3-II, Beclin-1, Atg3, and Atg5 demonstrated that the addition of forskolin significantly reduced radiation-induced autophagy at day 5 after treatment (*SI Appendix,* Fig. S2 *F* and *G*).

Reduced autophagy could influence tumor control in several ways. Autophagy is known to play a dual role in cancer, where it can promote cell survival under stress or, conversely, contribute to cell death. In the context of microglia-like cell differentiation, reduced autophagy might prevent excessive self-degradation and promote the functional stability of these cells resulting in a more sustained pro-inflammatory response and effective secretion of cytokines that hinder tumor growth.

Finally, we performed β-galactosidase staining to evaluate the presence of senescent cells after forskolin treatment. The analysis revealed a significantly increase in the number of senescent cells in response to forskolin, both with and without radiation, indicating that the combination of radiation and forskolin may reduce proliferation by promoting terminal differentiation (*SI Appendix*, Fig. S2 *H* and *I*).

### Bulk RNA Sequencing.

**Fig. 2.**
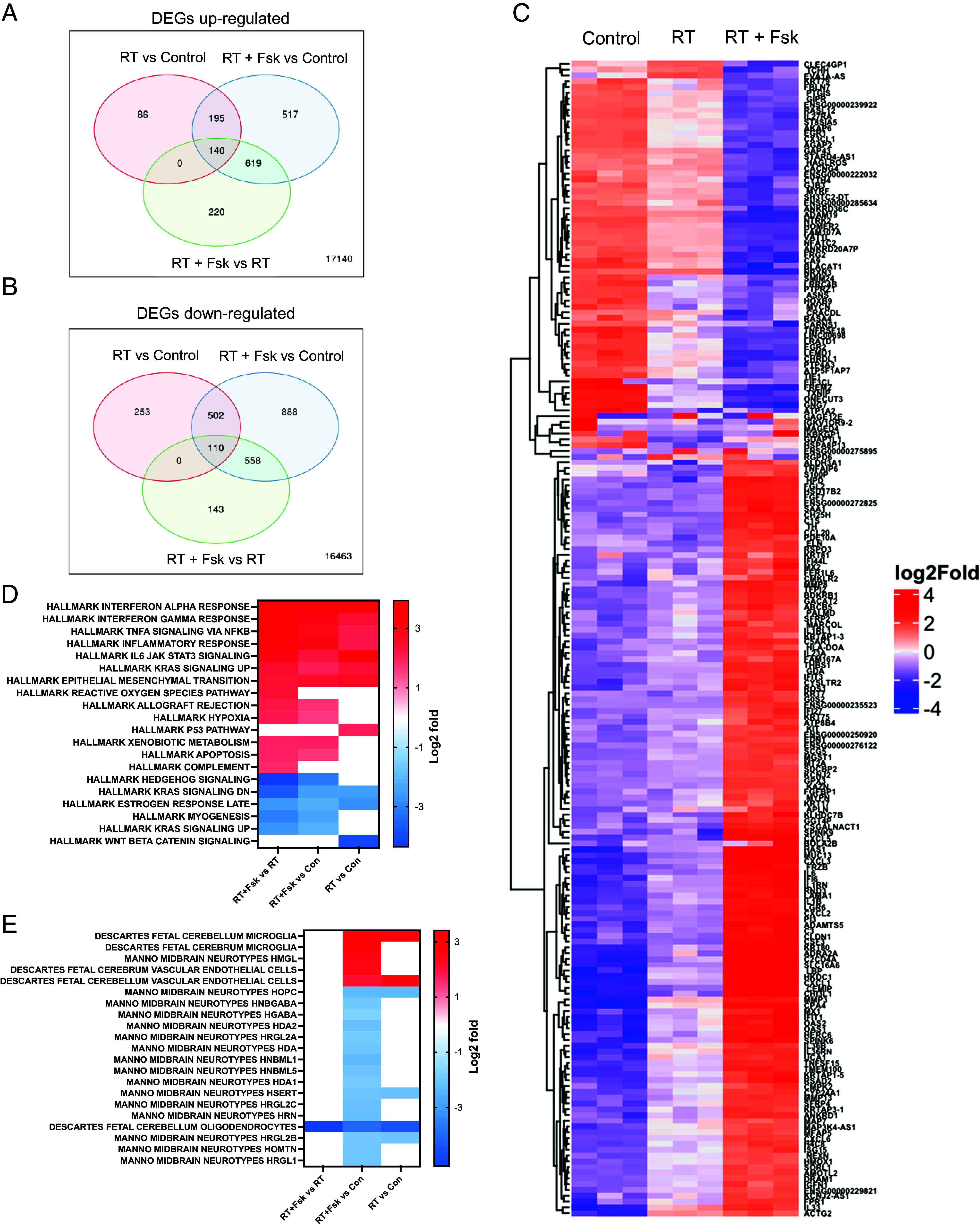
Bulk RNA-seq of cells treated with radiation and forskolin. Treatment with radiation and forskolin induced the upregulation (*A*) of 220 and downregulation (*B*) of 143 unique differentially expressed genes leading to a distinctive gene expression profile. Heatmap showing the top 200 differentially expressed genes (*C*). Enrichment analysis using the Hallmark.MSigDB gene set and the differentially expressed genes indicated enhancement of proinflammatory signaling after treatment with radiation and forskolin when compared to radiation alone, induction of reactive oxygen species, and inhibition of Shh and Kras signaling (*D*). Compared to unirradiated control cells the combination of radiation and forskolin led to gene expression profiles overlapping with those of microglia, neurons, and endothelial cells (Celltype.MSig.DB) (*E*). Abbrev. RT + Fsk represents RT + forskolin.

After observing the effects of increased neuronal differentiation in GBM cells in response to radiation combined with forskolin treatment, in order to assess which pathways were activated in cells irradiated and subsequently treated with forskolin and if surviving cells would display gene expression profiles associated with neuronal differentiation, we performed bulk RNA sequencing on HK-374 cells cultured as monolayers GSE285545 (24). Compared to unirradiated controls, cells irradiated with 4 Gy had 421 differentially expressed genes (DEGs) at least twofold (FDR 0.1) up- and 865 genes down-regulated. After combined treatment, we found 1,471 genes differentially up- and 2,058 genes down-regulated when compared to untreated control cells. Compared to irradiated cells, we found 945 DEGs up- and 704 genes down-regulated in cells treated with radiation and forskolin (Fig. 2 *A* and *B*), thus leading to a distinct gene expression profile for cells treated with radiation and forskolin (Fig. 2*C*).

Enrichment analysis of upregulated DEGs found in irradiated samples compared to unirradiated control cells using the Hallmark.MSigDB gene set revealed overlap with genes associated with proinflammatory pathways, epithelial-to-mesenchymal transition, and activation of the p53-dependent response (Fig. 2*D*), all consistent with the well-known effects of ionizing radiation (25). The inflammatory response was amplified by the addition of forskolin and suppression of genes in Hedgehog signaling.

Enrichment analysis of upregulated DEGs in cells treated with radiation and forskolin compared to control cells using the Celltype.MSigDB gene set showed overlap with genes associated with Manno Midbrain Neurotypes HMGL, Descartes Fetal Cerebrum and Cerebellum Microglia gene sets, as well as Descartes Fetal Cerebrum and Cerebellum Vascular Endothelial Cells while suppressing expression of genes associated with Manno midbrain neurotypes HOPC, HNBGABA, HDA1/2, HRGL2A, HAD, HNBML1/5 HSERT, HRGL2b/C, HRN, HOMTN, HRGL1, and Descartes fetal cerebellum oligodendrocytes (Fig. 2*E*).

### Single-Cell RNA Sequencing.

**Fig. 3.**
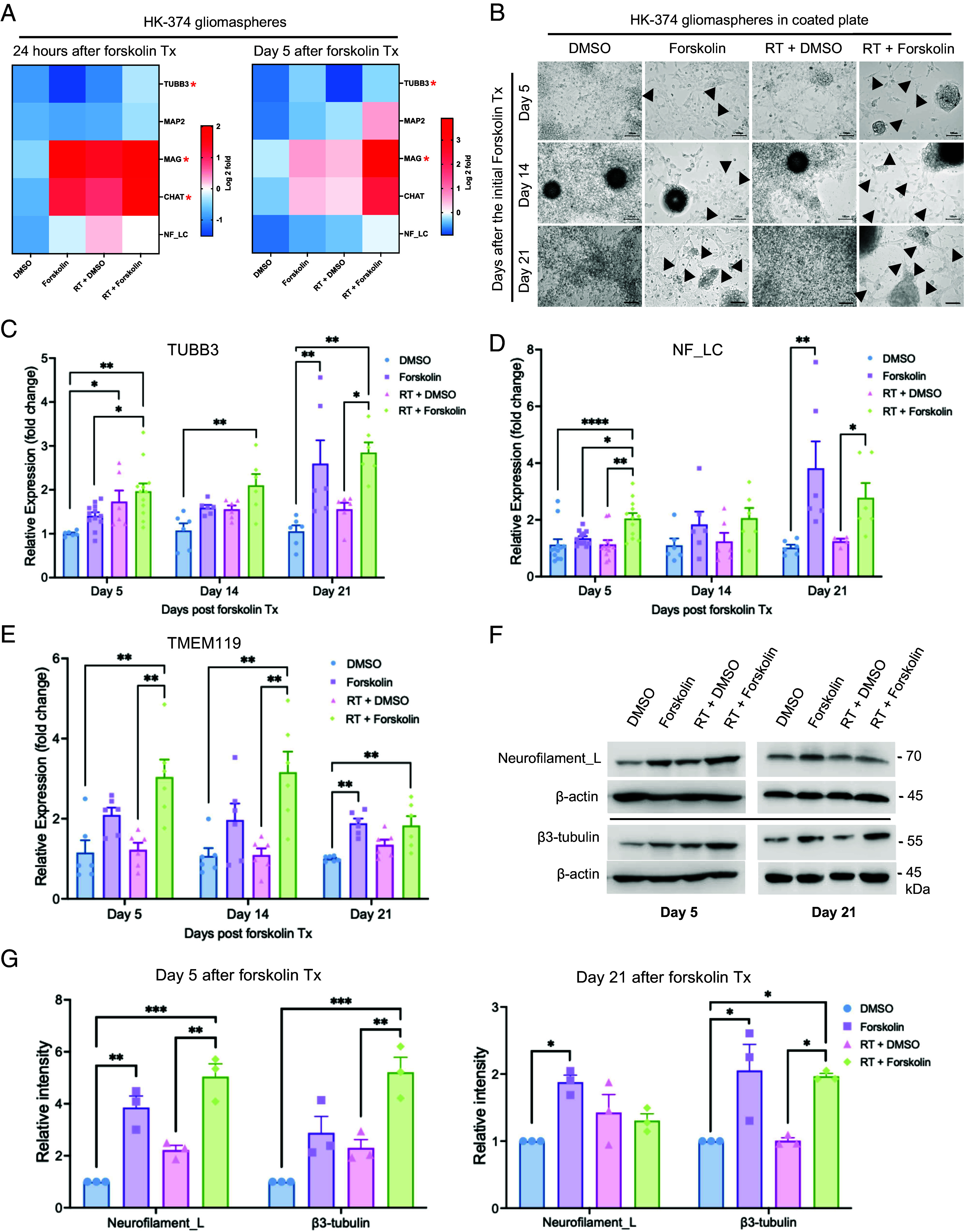
Glioma cells grown on poly-D-lysine/laminin-coated plates maintain the response to irradiation and forskolin treatment. Heatmaps showing the results of quantitative RT-PCR for the neuronal markers in HK-374 glioma spheres treated with radiation (a single dose of 4 Gy) in the presence or absence of forskolin (10 µM) for 24 h and 5 consecutive days under suspension culture in ultralow adhesion plates (*A*). Neuron-like cell morphology changes in HK-374 glioma spheres grown on Poly-D-Lysine/Laminin coated plates upon combined treatment of radiation and forskolin for 5, 14, and 21 d (black arrows showed the elongation of cell bodies) (*B*). Cells could be maintained under these culture conditions for 21 d with persisting significantly increased β3-tubulin, and NF_L marker expression after irradiation and forskolin treatment (*C* and *D*). Additionally, we found a significant increase of the microglia marker TMEM119 after treatment with radiation and forskolin (*E*). The significant increase in neuronal marker expression was confirmed in western blots (*F* and *G*). All experiments have been performed with at least three biological independent repeats (N = 3 to 6). *P-*values were calculated using one-way ANOVA for *C*–*E*, and *G*; The *P*-values listed in the heatmaps were from the comparison of RT + Forskolin to RT + DMSO. *P-value < 0.05, **P-value < 0.01, ***P-value < 0.001, and ****P-value < 0.0001.

Our qRT-PCR indicated that monolayer cultures, primarily consisting of more differentiated cells, increased neuronal marker expression in response to radiation combined with forskolin treatment. However, the effect was less pronounced when performed with gliomaspheres in suspension culture which are enriched for GSCs (*SI Appendix,* Fig. S3 *A* and *B*). We consider that the differentiated cells grown out from the surviving populations would die by anoikis under the suspension culture conditions (26). To allow for studying GSCs and more differentiated cells in parallel, we first tested whether gliomaspheres grown on Poly-D-Lysine/Laminin-coated plates would still respond to irradiation combined with forskolin treatment in similar ways as mostly differentiated monolayer cells. These culture conditions maintained viable cells for 21 d irrespective of the treatment arm (*SI Appendix,* Table S3). The addition of forskolin to unirradiated or irradiated cells induced neuronal marker expression (Fig. 3*A* and *SI Appendix*, Fig. S3 *C* and *D*) and morphological changes with elongated cell bodies (Fig. 3*B*, black arrows). qRT-PCR for neuronal markers revealed a significant increase in β3-tubulin expression (Fig. 3*C*), and NF_LC expression (Fig. 3*D*). Furthermore, we observed a significant increase in the expression of the microglia marker TMEM119 (Fig. 3*E*). Expression of neurofilament light chain (NF_L) and β3-tubulin at the protein level was confirmed by western blotting (Fig. 3 *F* and *G*).

**Fig. 4.**
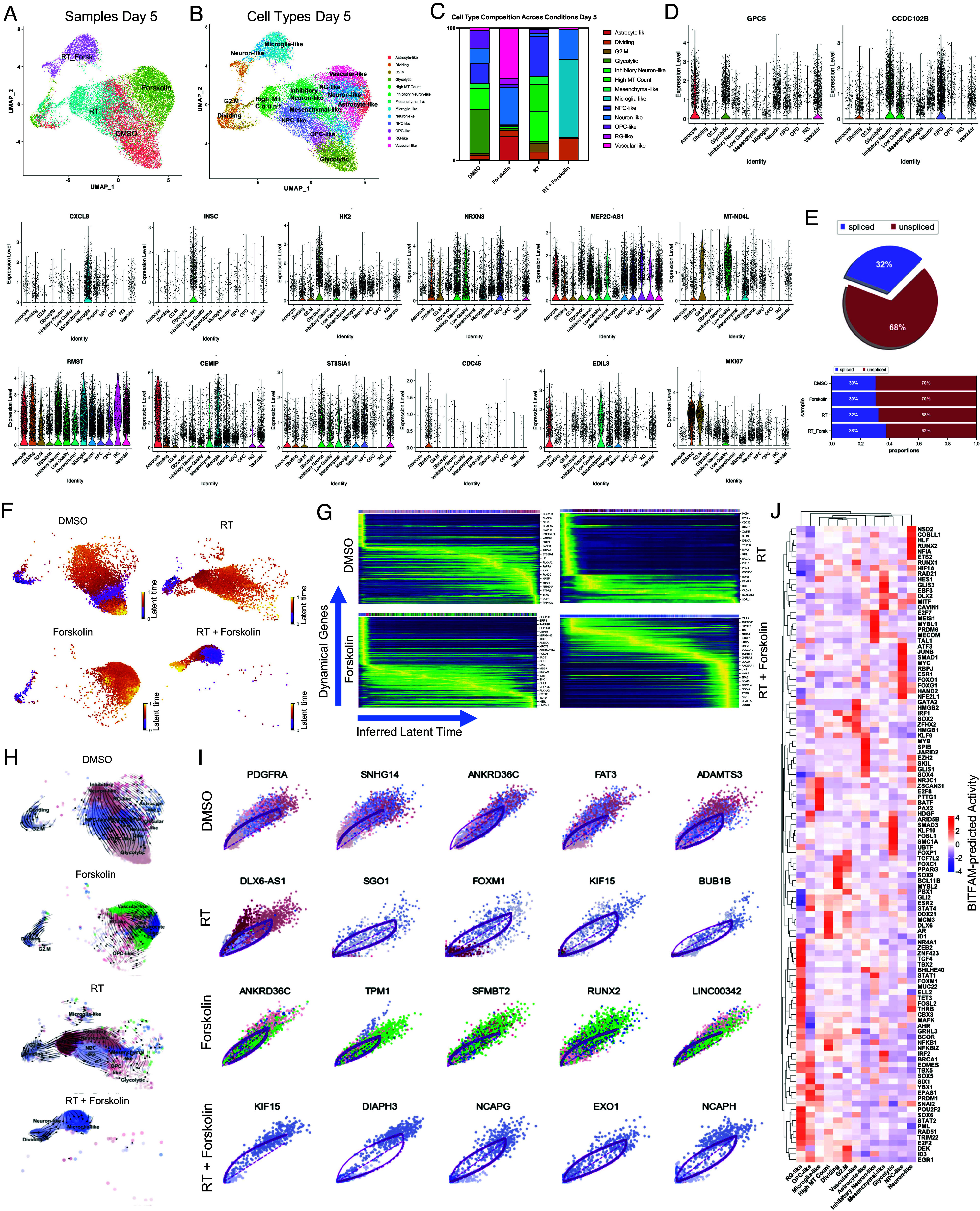
scRNA-seq of HK-374 glioma cells (day 5). UMAP plots (*A*) of 24 identified clusters that could be attributed to the different treatments. Cell type annotations are shown in *B*. Stacked columns show the distribution of the different cell types for each treatment condition (*C*). Violin plots of marker genes for the identified cell types are shown in *D*. Percentage of spliced and unspliced RNA in all samples and in each treatment condition (*E*). UMAP plot of latent time for all four conditions (*F*). Plot of dynamical genes against inferred latent time for all samples (*G*). Trajectory analysis for all four conditions (*H*). Expression of the top five driver genes for each condition over latent time (*I*). Transcription factor engagement for all predicted cell types calculated using a Bayesian model (BITFAM) (*J*).

Our bulk RNAseq data have shown that the gene expression profile in cells treated with radiation and forskolin overlapping with a gene set associated not only with a neuronal signature but also fetal microglia and vascular endothelial cells was rather unexpected, and we therefore sought to study differences in cell fate in more detail. We next performed scRNAseq using 20,028 cells treated with 0 (6,738) or 4 Gy (5,403), forskolin (5,494), or irradiation and forskolin (2,393), grown on Poly-D-Lysine/Laminin-coated plates for 5 d GSE285543 (27). Lovain clustering identified 24 unique clusters of cells that could be assigned to the different treatment arms of the study (*SI Appendix*, Fig. S4A). While there was some overlap between control cells (DMSO-treated) and irradiated cells and—to a lesser extent—control and forskolin-treated cells, cells treated with radiation and forskolin separated into 3 unique clusters (2, 17, and 18) (Fig. 4*A*). We next compared marker genes of the identified clusters to gene expression signatures of the adult and developing brain as described previously (28). Correlation coefficients were generally low, and the closest normal brain cell type was assigned to individual GBM clusters (Fig. 4*B*). The main clusters in untreated control cells were primarily composed of glycolytic- (33.4%), inhibitory neuron- (15.3%), NPC- (15.3%), OPC- (13%), and neuron-like cells (11.4%). 7 d after treatment with a single dose of 4 Gy the dominant cell populations were NPC-like cells (30.2%), inhibitory neuron-like (22.8%), and 22.8% of cells with high mitochondrial gene count (but less than 5%, which was our QC cutoff), the latter consistent with radiation-induced stress and cell death. In the current literature scRNAseq data of irradiated GBM cells are sparse, and the identity and role of these cells are unclear. But because these cells were cleared from the population at day 21, they most likely represent damaged cells that failed to divide, thus leading higher mitochondrial gene count.

The relative increase in NPC-like cells agreed with the known radioresistant phenotype of GSCs (3) and the decrease of the glycolytic-like cell population to 1.3% was in line with our previous reports of metabolic heterogeneity and metabolic plasticity (29, 30). Likewise, neuron-like and OPC-like cells dropped to 2% and 3.4%, respectively. After treatment with forskolin for five days, the dominant cell clusters were vascular-like cells (37.7%), neuron-like cells (28.3%), astrocyte-like cells (18.1%), and outer radial glia-like cells (oRG, 4.7%). Radiation combined with forskolin treatment resulted in the occurrence of microglia-like cells (59.1%) and neuron-like cells (22.7%), along with a population of cells in the G1/S phase of the cell cycle (‘Dividing,’ 16.2%). Additionally, there was a reduction in NPC-like cells (0.17%), OPC-like cells (0.22%), and oRG-like cells (0%) (*SI Appendix,* Table S4). Although microglia-like cells expressed immune-related genes they lacked expression of classical microglia markers. Notably, microglia-like cells were the rarest cell population in control cells and cells treated with radiation or forskolin alone (Fig. 4*C* and *SI Appendix,* Table S4). Violin plots of marker genes for the different cell types are shown in Fig. 4*D* and corresponding UMAP plots are shown in *SI Appendix,* Fig. S5. Distribution of the top five marker genes for microglia-like cells for all 4 conditions are shown in *SI Appendix,* Fig. S6.

Considering the notable shifts in cluster composition between the different treatments, we next sought to perform a trajectory analysis of the data based on RNA splicing information at day 5. Comparing the amount of spliced and unspliced RNA we found 70% of the RNA in control cells and forskolin-treated cells unspliced, while irradiated cells showed 68% of unspliced RNA. The amount of unspliced RNA dropped to 62% in cells treated with radiation and forskolin (Fig. 4*E*). Next, we used scVelo’s dynamical model to compute cell trajectories based on RNA expression and splicing information by calculating RNA velocities, dynamical genes, and latent time (31) for the three different treatments and the control sample cells that were used as starting point. UMAP plots of latent time suggested that control cells and cells treated with radiation or forskolin originated from dividing cells while cells treated with radiation and forskolin branched from the microglia-like cell population (Fig. 4*F*). We observed cascading dynamical genes over inferred latent time for all four conditions suggesting progression through differentiation of dedifferentiation steps (Fig. 4*G*), which was supported by the trajectory analysis of the cells (Fig. 4*H*). The trajectory analysis indicated dynamic conversion of cell types in untreated control cells consistent with the known intratumoral cellular heterogeneity of GBM. As expected, cells in the control sample developed into the different phenotypes from the dividing cell population. The main trajectory led to glycolytic cells from which a subset further developed into OPC-like, neuron-like, and NPC-like cells and into inhibitory neuron-like cells. This trajectory was maintained in irradiated cells, except for the occurrence of cells with high mitochondrial gene count originating directly from the dividing cell population. Treatment of the cells with forskolin caused a deviation from this trajectory with dividing cells transitioning into vascular-like cells, neuron-like cells, astrocyte-like cells, and outer radial glia-like cells (Fig. 4*H*).

Finally, treatment with radiation and forskolin completely altered the trajectory of the cells with the appearance of microglia-like cells and dividing cells leading into neuron-like cells, thereby omitting all other phenotypes (Fig. 4*H*). The top five driver genes for each condition are shown in Fig. 4*I*.

Next, we sought to identify transcription factors engaged in changes of gene expression profiles in individual cell types across all treatment groups using a Bayesian model (32) (Fig. 4*J*). For microglia-like cells the BITFAM algorithm calculated activity of PAX2, PTTG1, E2F8, NR3C1, YBX1, and BATF (activity cut-off of ≥ 2). None of these transcription factors are defining microglia cells.

For neuron-like cells, we calculated engagement of RUNX2, COBLL1, ETS2, HLF, NSD2, NFIA, and THRB, with the latter participating in CNS development (33).

**Fig. 5.**
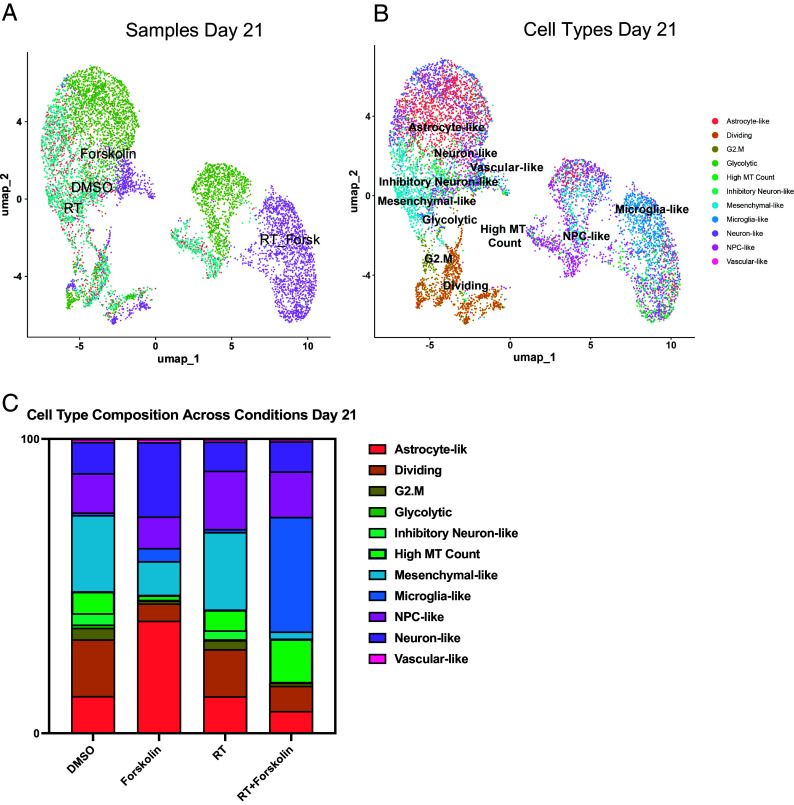
scRNA-seq of HK-374 glioma cells (day 21). UMAP plot of clusters identified 21 d after start of forskolin treatment attributed to the four different conditions (*A*) and projection of cell types identified on day 21 (*B*). Stacked columns show the distribution of the different cell types for each treatment condition (*C*).

Finally, we studied how stable these phenotypes would be over time. Repeating the scRNAseq experiment with cells treated for 21 d (25 unique clusters of cells were identified by Lovain clustering, *SI Appendix,* Fig. S4*B*) (34) GSE285544 and projecting cell types identified in day 5 samples onto clusters from day 21 (Fig. 5*A*), we observed that the culture conditions changed the overall composition of control cells with mesenchymal-like cells (25.8%), dividing cells (19.3%), NPC-like cells (13.4%), astrocyte-like cells (12.7%), or neuron-like cells (10.6%) as the dominant cell types and oRG-like and OPC-like cells no longer present in any of the treatment arms. At day 21, irradiated cells had largely redistributed to match the cluster distribution of control cells but still maintained an increase in NPC-like cells (19.8%). Forskolin-treated cells predominantly showed with astrocyte-like (38.2%), neuron-like (25.3%), mesenchymal-like (11.4%), or NPC-like (10.7%) phenotype. The dominant phenotypes in cells treated with radiation plus forskolin remained microglia-like cells (39%), followed by NPC-like (15.5%), and neuron-like cells (10.2%) (Fig. 5 B/C, *SI Appendix,* Table S5).

### A Combination of Radiation and Forskolin Improves Median Survival In Vivo.

**Fig. 6.**
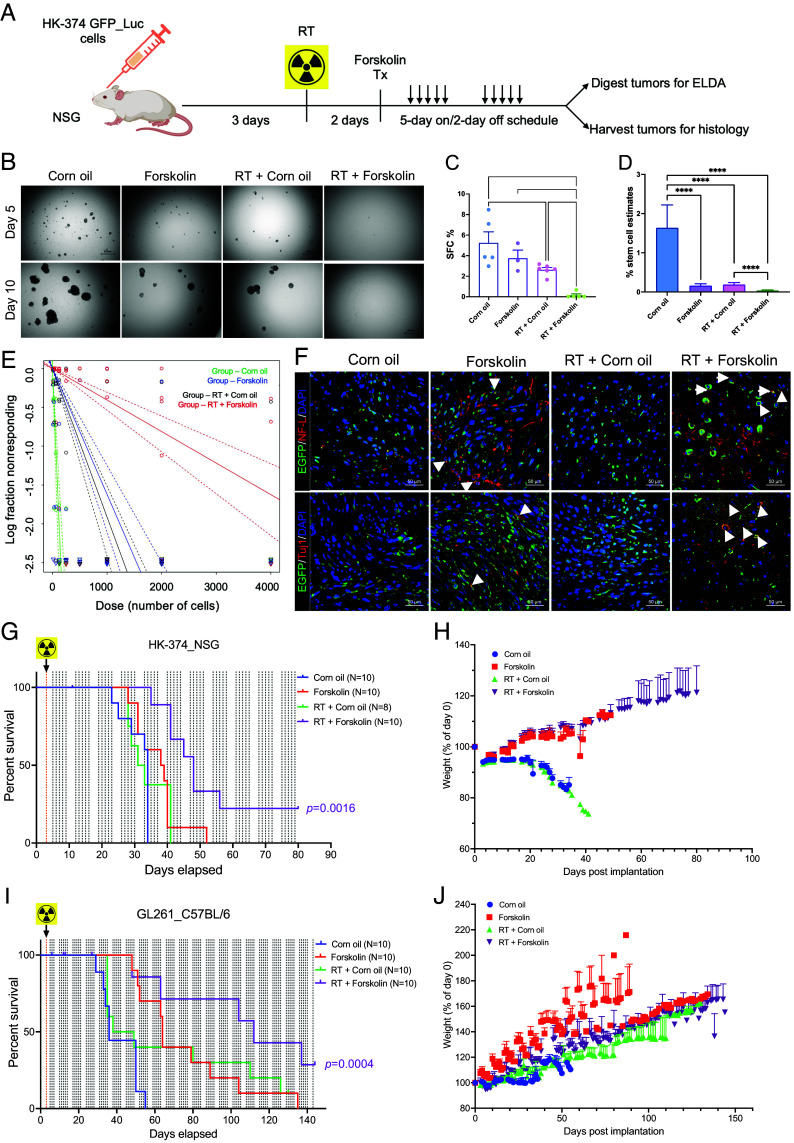
A combination of radiation and forskolin eliminates GSC in vivo and prolongs median survival in mouse models of glioblastoma (GBM). Schematic of the experimental design for Fig. 6 (*A*). Single-cell suspension from the tumors harvested showed a significant reduction in sphere formation for animals treated with radiation, while a combination of radiation and forskolin almost completely prevented sphere formation (*B* and *C*). Using an ELDA we found that forskolin treatment and irradiation alone significantly reduced the frequency of GSCs, while the combined treatment with radiation and forskolin nearly eliminated all GSCs (*D* and *E*). Confocal imaging of the tumor section of the animals revealed expression of NF_L and Tuj-1 in a subset of GFP-expressing tumor cells (*F*, white arrows: colocalization of double-stained cells). Treatment of HK-374 PDOXs in NSG mice (*G* and *H*) and intracranial syngeneic GL261 tumors in C57Bl/6 mice (*I* and *J*) with a single dose of 10 Gy and forskolin (5 mg/kg) was well tolerated and significantly prolonged the median survival of the animals. All experiments have been performed with at least three biological independent repeats (N = 3 to 10). *P-*values were calculated using one-way ANOVA for *C*; *P-*values were calculated and generated by ELDA software for *D*; log-rank (Mantel–Cox) test for comparison of Kaplan–Meier survival curves in *G* and *I*. *P-value < 0.05, **P-value < 0.01, and ***P-value < 0.001.

In silico, forskolin has an estimated LogBB of −0.24 (35), indicating its ability to cross the BBB (36). Therefore, we tested whether the combination of radiation and forskolin would affect the number of GSCs in vivo. HK374 cells were implanted into the striatum of NSG mice. After 3 d of grafting, tumors were irradiated with 0 or 4 Gy. After 48 h, the animals were treated with daily forskolin injections (5 mg/kg) on a 5-day on/2-day off schedule for 2 wk. Tumors were harvested, digested into single-cell suspensions, and subjected to clonal sphere-formation assays for 10 d (Fig. 6*A*). The combination of 4 Gy and Forskolin led to a significant reduction in sphere-forming capacity from 5.25% to 0.24% (*p*=0.015). Treatment with radiation alone also significantly reduced sphere formation, while forskolin alone had no significant effect (Fig. 6 B and C). Next, we calculated GSC frequencies using an ELDA. Both irradiation and forskolin treatment significantly reduced the number of GSCs, while the combination of radiation and forskolin significantly reduced GSC numbers to 0.04%, 95% CI: 0.03% to 0.05%, *P* < 0.0001 (Fig. 6 *D* and *F* and *SI Appendix,* Tables S6 and S7). Confocal imaging of corresponding tumor sections revealed that forskolin treatment and combined treatment with radiation and forskolin induced the expression of NF_LC and Tuj1 in GFP-expressing GBM cells in vivo (Fig. 6*F*, white arrows).

Finally, we tested whether the combination of radiation and forskolin would lead to an improved median survival in mouse models of glioma. HK374 cells were implanted into the striatum of NSG mice. After 3 d of grafting, patient-derived orthotopic xenografts (PDOXs) were irradiated with a single dose of 10 Gy, equivalent to a total dose of 18 Gy in 2 Gy fractions. Control mice were sham irradiated. Forty-eight hours later, mice were treated with forskolin at 5 mg/kg until they reached the criteria for being killed. Control animals were treated with solvent only. While radiation or forskolin treatment alone had no effect in this highly aggressive and rapidly growing PDOX, the combination of radiation and forskolin significantly increased the median survival of the animals from 34 to 48 d (Fig. 6*G*; *P* < 0.0001, log-rank test). Forskolin treatment was well tolerated, and animals continued to gain weight after combined treatment (Fig. 6*H*).

In the syngeneic GL261 glioma mouse model, control mice had a median survival of 36 d. Median survival was increased to 43.5 d in irradiated mice (not significant), to 64 d in mice treated with forskolin (*P* = 0.001, log-rank test) and to 129 d in mice receiving the combination of radiation and forskolin (Fig. 6*I*; *p*=0.0021, log-rank test). As in NSG mice, the addition of forskolin was well tolerated (Fig. 6*J*).

## Discussion

In our current study, we hypothesized that glioma cells, that survive irradiation, go through a transient state of multipotency that can be exploited to drive GBM cells into a postmitotic neuron-like state. Our hypothesis was based on our previous observations that differentiated nonstem glioma cells that survive exposure to ionizing radiation respond with a phenotype conversion into induced GSCs (15), while surviving, preexisting GSCs transdifferentiated into endothelial- and pericyte-like cells (18). The underlying mechanisms were global epigenetic remodeling through changes in histone methylation and acetylation and subsequent changes in open chromatin (15, 18), with the latter mediated by the histone acetyltransferase EP300 (18). Induced GSCs and transdifferentiated pericyte- and endothelial-like cells contributed to tumor recurrence and treatment resistance (15, 18) and preventing phenotype conversion or transdifferentiation prolonged median survival in PDOXs mouse models of GBM (15, 18, 37, 38). Here, we have shown that the addition of forskolin to radiation drives GBM cells into microglia- and neuron-like states, reduced cell proliferation, and the number of functional GSCs in vitro and in vivo and prolonged the median survival in syngeneic and PDOX mouse models of GBM.

It is important to note that microglia are of mesodermal origin, whereas glioma stem-like cells (GSCs) are thought to closely resemble ectodermal neural stem cells (NSCs) or oligodendrocyte progenitor cells (OPCs). However, under certain conditions, particularly within the tumor microenvironment, trans-differentiation between different cellular lineages, including ectodermal and mesodermal origins can occur, allowing tumor cells to adapt to environmental pressures (39, 40) and in a recent study we reported the acquisition of mesenchymal features in irradiated GBM cells (18).

Several previous studies have attempted differentiation therapies for GBM. Most studies succeeded to some extent in vitro with cells showing decreased tumorigenicity when implanted into mice. For example, all-trans retinoic acid (ATRA) successfully differentiated GSCs in vitro and reduced their tumorigenicity in vivo (41) but clinical translation targeting GBM with ATRA has consistently failed (42, 43). Another study induced GSC differentiation through stereotactical injection of a WNT inhibitor, an SHH inhibitor, and BMP (44). While effective, the intracranial application route could be clinically challenging and is likely to miss GBM cells, dispersed into the normal parenchyma that are not in reach of the injections.

Forskolin and cAMP have long been known to induce glioma differentiation in vitro (45, 46) and the use of forskolin or dbcAMP to induce terminal neuronal differentiation of glioma in vivo has been previously reported (47). Like in our current study, this treatment alone had minimal effects on median survival even when combined with a Wnt inhibitor and even though dbcAMP was given at a high dose (47). Our scRNAseq data indicate that forskolin treatment alone not only induced neuron-, astrocyte-, and vascular-like cells but also a small number of cells with an outer radial glia-like cell state (4.7%), a cell population present in small numbers in control and irradiated cells but completely absent in cells treated with radiation and forskolin. It was previously reported that oRG cells have GSC features (28), and this observation could explain why dbcAMP or forskolin treatment alone has limited impact on median survival in glioma models.

Radiation therapy is and—for the foreseeable time—will remain the most effective treatment modality against GBM, and any novel treatment modality will most likely be added to the standard of care, surgery and radiation, or will at least be benchmarked against it. Our finding that radiation induces a transient multipotent state added a facet to the radiation response of cancer, that leads to increased cellular plasticity, which is increasingly recognized as an emerging hallmark of cancer (48). This increase in plasticity leads to dedifferentiation or transdifferentiation and is by itself detrimental with respect to tumor control. By exploiting radiation-induced multipotency, our approach highjacks a unique feature of surviving cells that is inevitably induced by a main pillar of the current standard of care. With the addition of forskolin to radiation treatment, we utilize an established method that forces iPS cells into neuronal differentiation.

GBM cells acquire a certain degree of multipotency, whereas iPS cells display varying levels of multipotency, with significant differences in mutational burden. Additionally, irradiated GBM cells persist in signaling through the DNA damage response. Hence, it is not expected that forced differentiation of GBM cells will yield in bona fide induced neurons. Our finding that the combination of radiation and forskolin predominately led to the occurrence of microglia-like cells and to a lesser extent to neuron-like cells was unexpected. The role of this predominant immune cell-like phenotype in the presence of an intact immune system needs further investigation. However, a combination of radiation and forskolin was superior to their individual effects on inducing neuronal marker expression and inhibiting cell proliferation of GBM cells, reducing self-renewal capacity of GSCs and GSC frequency and prolonging median survival of glioma-bearing mice and leading to long-term tumor control in some of the mice. This suggested that this combination therapy induced a cell phenotype that could no longer sustain tumor growth. With forskolin crossing the BBB and its long use in ayurvedic medicine, this combination therapy can be easily translated into a clinical trial.

However, our study has limitations. Even with continuous forskolin treatment, the tumor-bearing mice eventually experienced a decline, indicating that while the initial response is promising, the long-term efficacy may be limited. This suggests that the study’s scope primarily addresses short- to medium-term outcomes without fully evaluating the potential long-term effects or sustainability of forskolin treatment. Another possibility is that the very low dose of forskolin used here is not optimal and dose escalation studies will be necessary to maximize the effect.

Additionally, the implications of chronic forskolin administration on normal brain tissue and its interactions with glioma cells remain uncertain. Continuous treatment may present challenges related to potential toxicity, the development of resistance, or alterations in the tumor microenvironment over time. These aspects were not thoroughly examined in the current study, highlighting a need for further investigation into the biological and clinical feasibility of sustained forskolin use.

Future research should aim to explore the long-term durability of tumor response and consider whether alternative dosing strategies, such as intermittent or optimized regimens, could offer comparable or superior benefits while minimizing potential risks. This could help in identifying an effective balance between treatment efficacy and safety, ensuring that the benefits of forskolin treatment can be maintained without adverse long-term effects.

## Materials and Methods

This section provides an overview of the principal materials and experimental methods employed in the study. Detailed protocols, including those related to bulk and single-cell RNA sequencing workflows, PDOX and syngeneic mouse models of GBM, irradiation procedures, pharmacological interventions, as well as methods for γH2AX foci detection and senescence analyses, are presented in *SI Appendix*. The appendix also includes additional technical information regarding data processing, quality control, statistical analyses, and any specialized equipment or reagents used throughout the experiments.

### Cell lines, PDOX, and Syngeneic GBM Mouse Models.

Primary human glioma cell lines were established at UCLA as described in ref. 1. All GBM lines were deidentified before use, and their use is covered by UCLA’s Institutional Review Board protocol IRB#16-001351. Characteristics of specific gliomasphere lines can be found in ref. 49. The GL261 murine glioma cell line was a kind gift of Dr. William H. McBride (Department of Radiation Oncology at UCLA). All cell lines were routinely screened for *Mycoplasma* contamination.

For in vivo experiments, GFP luciferase tagged cells were implanted into the right striatum of mouse brains using a stereotactic frame and a nano-injector pump. Tumors were allowed to grow for three days, and successful engraftment was confirmed by bioluminescence imaging before initiating treatments.

### Bulk and Single-Cell RNA Sequencing.

Patient-derived GBM cell cultures were treated with forskolin—a known cyclic AMP pathway modulator—either with or without concurrent radiation exposure. Following the designated treatment period, cells were prepared for transcriptomic analysis through both bulk and single-cell RNA sequencing (scRNA-seq) methodologies.

## Supplementary Material

Appendix 01 (PDF)

## Data Availability

All sequencing data have been deposited in Gene Expression Omnibus (GSE285543 (27), GSE285544 (34), and GSE285545 (24)). All study data are included in the article and/or *SI Appendix*.
