# Supplementary material for: Radiation-induced cellular plasticity primes glioblastoma for forskolin-mediated differentiation
Source: Proc Natl Acad Sci U S A. 2025 Feb 26;122(9):e2415557122. doi: 10.1073/pnas.2415557122 (PMC11892679; doi:10.1073/pnas.2415557122)
Supplement: Supplementary file 1 — Appendix 01 (PDF) [file pnas.2415557122.sapp.pdf]

Supplementary Materials

Radiation-Induced Cellular Plasticity Primes Glioblastoma for Forskolin-Mediated Differentiation

[1,2]Ling He, D.D.S. Ph.D., [3]Daria Azizad, [1]Kruttika Bhat, Ph.D., [1]Angeliki Ioannidis, M.S., [1]Carter J. Hoffmann, M.S., [1]Evelyn Arambula, [4]Mansoureh Eghbali, Ph.D., [2, 3]Aparna Bhaduri, Ph.D., [2, 5]Harley I. Kornblum, M.D., Ph.D and [1,2,6]Frank Pajonk, M.D., Ph.D*

Table of contents:

1. **Supplementary Materials and Methods**
2. **Supplementary Figures**
3. **Supplementary Tables**
4. **References**

Supplementary Materials and Methods

Cell Culture

Primary GBM cells were propagated as gliomaspheres in serum-free conditions in ultra-low adhesion plates in DMEM/F12, supplemented with SM1 Neuronal Supplement (#05177, STEMCELL Technology, Kent, WA), EGF (#78006, STEMCELL Technology), bFGF (#78003, STEMCELL Technology) and heparin (1,000 USP Units/mL, NDC0409-2720-31, Lake Forest, IL) as described previously (1-3). GL261 cells were cultured in log-growth phase in DMEM supplemented with 10% fetal bovine serum, penicillin, and streptomycin. All cells were grown in a humidified atmosphere at 37°C with 5% CO_2. The unique identity of all patient-derived specimens was confirmed by DNA fingerprinting (Laragen, Culver City, CA). All lines were routinely tested for mycoplasma infection (#G238, Applied biological Materials, Ferndale, WA).

Animals

Female 6–8-week-old C57BL/6 mice, or NOD-*scid* IL2Rgamma^{null} (NSG) originally obtained from The Jackson Laboratories (Bar Harbor, ME) were re-derived, bred and maintained in a pathogen-free environment in the American Association of Laboratory Animal Care-accredited Animal Facilities of Department of Radiation Oncology at UCLA. For intracranial implantation, $2x10^5$ GL261-Luc or $3x10^5$ HK-374-Luc cells were implanted into the right striatum of the brains of mice using a stereotactic frame (Kopf Instruments, Tujunga, CA) and a nano-injector pump (Stoelting, Wood Dale, IL). Injection coordinates were 0.5 mm anterior and 2.25 mm lateral to the bregma, at a depth of 3.0 mm from the surface of the brain. Tumors were grown for 3 days with successful grafting confirmed by

bioluminescence imaging. Weight of the animals was recorded daily. Mice that lost 20% of their body weight or developed neurological deficits requiring euthanasia were sacrificed.

Ethics statement

All animal experiments were approved by UCLA's Institutional Animal Care and Use Committee in accordance with all local and national guidelines for the care of animals.

Extreme Limiting Dilution Analysis (ELDA)

HK-374 cells were intracranially implanted into the NSG mice as described above. Tumors were grown for 3 days for successful grafting. Tumor-bearing mice were then irradiated with a single dose of 4 Gy and injected intra-peritoneally on a 5-days on / 2-days off schedule for 2 weeks either with corn oil or forskolin starting 48 hours after the irradiation. The mice were then euthanized and tumor-bearing brains were dissected and further subjected for dissociation using mouse Tumor Dissociation Kit (# 130-096-730, Miltenyi, Auburn, CA) to get single cell suspension, as described in (4). The cells were counted and plated into the non-tissue-culture-treated 96-well plates at a range of 1 to 512 cells/well. Growth factors (EGF and bFGF) were supplemented every two days. Glioma spheres were counted 10 days later and presented as the percentage to the initial number of cells plated. The glioma stem cell frequency was calculated using the ELDA software (5).

Western Blotting

HK-374 cells were plated and irradiated the next day with 4 Gy. 48 hours after the irradiation, cells were daily treated with forskolin (10 μM) for 5 days or 21 days. The cells were then lysed in RIPA lysis buffer containing proteinase inhibitor and phosphatase inhibitor. 150 μl of ice-cold RIPA lysis buffer (10 mM Tris-HCl (pH 8.0), 1 mM EDTA, 1% Triton X-100, 0.1% Sodium Deoxycholate, 0.1% SDS, 140 mM NaCl, 1 mM PMSF) containing proteinase inhibitor (Thermo Fisher Scientific) and phosphatase inhibitor (Thermo Fisher Scientific) was used to lyse the cells. The protein concentration in each sample was determined by BCA protein assay (Thermo Fisher Scientific) and samples were denaturated in 4x Laemmli sample buffer (Bio-Rad) containing 10% β-mercaptoethanol for 10 minutes at 95 °C. Equal amounts of protein were loaded onto 10% SDS-PAGE gels and subjected to electrophoresis for 2 hours. Samples were then transferred onto 0.45 μm nitrocellulose membrane (Bio-Rad) and blocked in 1x TBST containing 5% bovine serum albumin (BSA) for 30 minutes at RT, followed by incubation with primary antibodies against Neurofilament-L (#2837S, 1:1000, Cell Signaling Technology), GFAP (#12389S, 1:1000, Cell Signaling Technology), β3-tubulin (#5568S, 1:1000, Cell Signaling Technology), Atg3 (#3415, 1:1000, Cell Signaling Technology), Atg5 (#12994, 1:1000, Cell Signaling Technology), Beclin-1 (#3495, 1:1000, Cell Signaling Technology), LC3A/B (#12741, 1:1000, Cell Signaling Technology) and β-actin (#3700S, 1:1000, Cell Signaling Technology) in 1X TBST containing 5% BSA overnight at 4°C with gentle rocking. Membranes were then washed three times for 5 minutes each with 1X TBST and incubated with secondary antibodies, 1:5000 anti-mouse or anti-rabbit horseradish peroxidase (HRP; Cell Signaling) in 1X TBST for two hours at RT with gentle rocking. Membranes were washed again three times for 5 minutes each with 1X TBST.

Pierce ECL Plus Western Blotting Substrate (Thermo Fisher) was added to each membrane and incubated at RT for 5 minutes. The blots were then scanned using the Odyssey Fc imaging system (LI-COR Biosciences, Lincoln, NE). β-actin was used as a loading control. Densitometry was performed using ImageJ. The ratio of the protein of interest over its endogenous control was calculated and expressed as relative intensity.

Immunofluorescence

For *in vitro* neuronal marker staining, HK-374 cells were trypsinized and plated onto the round glass coverslips at a density of 2 x 10^4 cells/well. The following day, cells were irradiated with 4 Gy and then daily treated with forskolin (10 μM) for 5 days starting 48 hours after irradiation. For *in vivo* EGFP and neuronal marker co-staining, mouse brains were explanted, fixed in formalin and embedded in paraffin. Immunofluorescence staining was performed on these paraffin-embedded slides.

At day 5, the coverslips were fixed in formalin at RT for 15 minutes and washed three times with PBS, then permeabilized by 0.5% Triton X-100 for 10 minutes at room temperature. After three-time PBS washing, the coverslips were blocked with 10% goat serum diluted in PBS for 1 hour at RT and then incubated with primary antibodies against Neurofilament-L (Cell Signaling Technology, #2837, 1:100), and β3-tubulin (Cell Signaling Technology, #5568s, 1:400) overnight at 4°C. The next day, the secondary antibodies Alexa Fluor 594 Goat Anti-rabbit immunoglobulin G (IgG) (H/L) antibody (1:1,000 (Invitrogen)) or Alexa Fluor 488 Goat Anti-rabbit IgG (H/L) antibody (1:1,000 (Invitrogen)) were applied for 60 min, with subsequent nuclear counterstaining with Hoechst 33342 (Invitrogen, Cat# H3570, 1:5000). The sections were sealed with

VECTASHIELD® PLUS Antifade Mounting Medium (Vector Laboratories, Cat#H-1900) and images were taken with a digital microscope (BZ-9000, Keyence, Itasca, IL).

For γH2AX staining, coverslips were fixed, permeabilized, and blocked with 10% goat serum in PBS for 1 hour at RT, one and twenty-four hours after forskolin treatment. The γH2AX primary antibody (Cell Signaling Technology, #9718s, 1:250) was then applied and incubated overnight at 4°C. The remaining staining procedures were performed as previously described.

For *in vivo* EGFP and neuronal marker co-staining, brain sections were baked for 30 min in an oven at 65 °C, deparaffinized in two successive Xylene baths for 5 minutes each and then hydrated for 5 minutes each using an alcohol gradient (ethanol 100%, 90%, 70%, 50%, 25%). Antigen retrieval was performed using Heat Induced Epitope Retrieval in a citrate buffer (10 mM sodium citrate, 0.05% tween20, pH 6) with heating to 95 °C in a steamer for 20 minutes. After cooling down, the slides were blocked with 10% goat serum plus 1% BSA at RT for 30 minutes and then incubated with the primary antibodies against EGFP (Abcam, ab184601, 1:100) mixed with Neurofilament-L (Cell Signaling Technology, #2837, 1:100) or EGFP (Abcam, ab184601, 1:100) mixed with β3-tubulin (Cell Signaling Technology, #5568s, 1:400) overnight at 4°C. The secondary antibodies Alexa Fluor 594 Goat Anti-rabbit immunoglobulin G (IgG) (H/L) antibody (1:1,000 (Invitrogen)) and Alexa Fluor 488 Goat Anti-mouse IgG (H/L) antibody (1:1,000 (Invitrogen)) were applied followed by nuclear counterstaining and mounting procedures

as above. Fluorescent images were then acquired using a confocal microscope (Nikon A1, Melville, NY).

cAMP Assay

HK-374 monolayers were trypsinized and plated at a density of 5 x 10^4 cells/well in surfaced treated 96-well plates, while the HK-374 gliomaspheres were dissociated and plated at the same density in non-treated 96-well plates. The following day, cells were treated with freshly prepared forskolin (#F3917, Sigma, St. Louis, MO) at 0.1, 0.25, 0.5, 1, 5, 10, 50, and 100 µM concentrations, with DMSO serving as the solvent control. 15 minutes after the treatment, the adherent monolayers were incubated with 100 µl/well cell lysis buffer from the cAMP Direct Immunoassays Kit (Fluorometric, ab138880, Abcam, Cambridge, UK), while the gliomaspheres were collected, centrifuged down and further incubated with 100 µl cell lysis buffer at RT for 10 minutes. 25 µl cell lysates were used to quantify the cAMP concentration by comparing to the standard cAMP curve. All procedures were performed following the manufacturer's guidelines.

Quantitative Reverse Transcription-PCR

Total RNA was isolated using TRIZOL Reagent (Invitrogen, Waltham, MA). cDNA synthesis was carried out using the SuperScript Reverse Transcription IV (Invitrogen). Quantitative PCR was performed in the QuantStudioTM 3 Real-Time PCR System (Applied Biosystems, Carlsbad, CA, USA) using the PowerUpTM SYBRTM Green Master Mix (Applied Biosystems). Ct for each gene was determined after normalization to PPIA and ΔΔCt was calculated relative to the designated reference sample. Gene

expression values were then set equal to $2^{-\Delta\Delta Ct}$ as described by the manufacturer of the kit (Applied Biosystems). All PCR primers were synthesized by Invitrogen. PPIA was used as a housekeeping gene.

Irradiation

Cells were irradiated at RT using an experimental X-ray irradiator (Gulmay Medical Inc. Atlanta, GA) at a dose rate of 5.519 Gy/min. Control samples were sham-irradiated. The X-ray beam was operated at 300 kV and hardened using a 4 mm Be, a 3 mm Al, and a 1.5 mm Cu filter and calibrated using NIST-traceable dosimetry. Corresponding controls were sham irradiated.

For *in vivo* irradiation experiments, mice were anesthetized prior to irradiation with an intra-peritoneal injection of 30 µL of a ketamine (100 mg/mL, Phoenix, MO) and xylazine (20 mg/mL, AnaSed, IL) mixture (4:1) and placed on their sides into an irradiation jig that allows for irradiation of the midbrain while shielding the esophagus, eyes, and the rest of the body. Animals received a single dose of 10 Gy on day 3 after tumor implantation.

Drug treatment

For *in vitro* studies, HK374, HK308 or HK157 cells were plated to form monolayers or gliomaspheres and irradiated at a single dose of 4 Gy the next day. 48 hours after irradiation, cells were treated with dibutyryl-cAMP (dbcAMP; #D0627, Sigma), a cell-permeable analog of cyclic adenosine 3'5'-monophosphate (cAMP), at 1mM or forskolin at 10 µM for 5 consecutive days.

For *in vivo* studies, tumor grafting was confirmed via bioluminescence imaging. Mice implanted with either HK374 cells or GL261 cells were injected intraperitoneally with forskolin (#F3917, Sigma) at 5 mg/kg on a 5-days on / 2-days off schedule starting 48 hours after irradiation until they reached euthanasia endpoints. Forskolin was dissolved in corn oil containing 2.5% DMSO at a concentration of 0.55 mg/ml and prepared freshly for the injections.

Cell cycle and G_0 phase analysis

Following treatment with 10 μM forskolin, HK-374 cells were trypsinized, rinsed with ice-cold PBS, and fixed in cold 70% ethanol while gently vortexing. The cells were centrifuged at 500 x g for 4 minutes, resuspended in 200 μl UltraPure RNase (Thermo Fisher, #12-091-021), and transferred to FACS tubes. Next, 200 μl of Propidium Iodide solution (1 mg/ml, Thermo Fisher, #P1304MP) was added, and the cells were incubated for 15 minutes at room temperature in the dark. Flow cytometry was performed on at least 100,000 events using an LSR Fortessa (BD, San Jose, CA), and data were analyzed with FlowJo v10.

For G_0 phase analysis, after fixation in 70% ethanol, DNA was stained with 1 μg/ml Hoechst 33342, and RNA was stained with 2 μg/ml Pyronin Y (PY) in PBS for 30 minutes at RT in the dark. The cells were then washed and resuspended in PBS. Flow cytometry analysis was conducted on at least 100,000 cells, with Hoechst 33342 detected in the BV421 channel and PY in the PE channel.

γH2AX FACS Analysis

HK-374 monolayer cells were seeded in 10-cm dishes and subjected to a single 4 Gy radiation dose the following day. Forty-eight hours post-irradiation, cells were treated with 10 μM forskolin. One and twenty-four hours after treatment, cells were trypsinized and fixed in 4% paraformaldehyde (PFA) for 20 minutes at RT, followed by permeabilization using ice-cold 100% methanol on ice for 10 minutes. The cells were then washed and stained with a γH2AX antibody (Cell Signaling Technology, #9718s, 1:250) for 1 hour at RT, followed by incubation with Alexa Fluor 488 Goat Anti-rabbit IgG (H/L) secondary antibody (1:500, Invitrogen) for 30 minutes at RT. Finally, the cells were washed, resuspended in PBS, and analyzed by flow cytometry, with a minimum of 100,000 cells collected.

Senescence β-Galactosidase Staining

HK-374 monolayer cells were seeded into a 12-well plate at a density of 5,000 cells per well and irradiated with a single dose of 4 Gy the following day. Forty-eight hours post-irradiation, the cells were treated with 10 μM forskolin continuously for 5 days. On day 5, the cells were fixed and washed with PBS, after the last wash, staining solution was added [1 mg/ml 5-bromo-4-chloro-3-inolyl-β-D-galactoside (X-gal) (20 mg/ml stock, in dimethyformamide), 40 mM citric acid/sodium phosphate, pH 6.0, 5 mM potassium ferrocyanide, 5 mM potassium ferricyanide, 150 mM NaCl, 2 mM $MgCl_2$] and cells were incubated in a 37°C for 18 hours. After incubation, cells were washed two times with PBS and visualized with a digital microscope (BZ-9000, Keyence, Itasca, IL). The percentage of β-galactosidase-positive cells was counted and quantified with Image J.

Bulk RNA sequencing

HK-374 cells were seeded into 6-well plates as monolayer cultures at 40,000 cells/well. 4 days after seeding the cells were irradiated with 4 Gy. Controls were sham irradiated. 48 hours later cells were treated with either forskolin or DMSO for 5 consecutive days. Total RNA was isolated using Trizol. Bulk RNA-seq was performed by Novogene and reads were mapped to the human genome (hg38) following their standard pipeline (4). Read counts were analyzed using the iDEP package (version 2.0) (6). Differentially expressed genes were calculated using the DESeq2 algorithm with a minimum of a 2-fold change and a false discovery rate (FDR) of 0.1. Enrichment *p*-values were calculated based on a one-sided hypergeometric test. *P*-values were then adjusted for multiple testing using the Benjamini-Hochberg procedure and converted to FDR. Fold Enrichment was defined as the percentage of genes in the list belonging to a pathway, divided by the corresponding percentage in the background.

Single Cell RNA Sequencing

HK-374 gliomaspheres were plated onto the Poly-D-Lysine/Laminin 6-well plate (#354595, Corning) and irradiated with 4 Gy the next day. 48 hours after the irradiation, the cells were treated with forskolin (10 μM) for 5 consecutive days or 21 days. At day 5 or day 21, the gliomaspheres in the suspension culture were collected and dissociated with TrypLE (no phenol red, Thermo Fisher Scientific), and the adherent differentiated cells were washed with HBSS and de-attached with Trypsin/EDTA (Cell Applications, Cat#090K). The cells were then pooled and filtered through a 40-μm strainer and fixed

with Evercode^{TM} Cell Fixation v2 kit (#ECF2101, Parse Biosciences, Seattle, WA) following the manufacturer's guidelines. The samples (n=3 for each condition) were then sent out to the Genomics High Throughput Facility (GHTF) at the University of California, Irvine for subsequent single cell RNA sequencing (scRNA-seq) using the Evercode^{TM} Whole Transcriptome Mini kit (#EC-W01010, Parse Biosciences). Sequencing reads from the mRNA libraries were mapped to the human genome (hg38) using the Parse Biosciences pipeline (split-pipe vers. 1.1.1) to generate cell by gene counts matrices. Data analysis was performed using the R package Seurat (version 4.3.3). Matrices were filtered for cells with high mitochondrial and ribosomal gene count and doublets were removed using DoubletFinder R package. For the subsequent cluster annotation, we used three published gene sets associated with cell types in the developing brain as previously described(7). The trajectory analysis was executed using the Python package scVelo (version 0.2.5)(8). Engagement of transcription factors was determined using the R package BITFAM(9).

Statistics

All data shown are represented as mean ± standard error mean (SEM) of at least 3 biologically independent experiments. A *p*-value of ≤0.05 in an unpaired two-sided *t*-test or one-sided ANOVA for multiple testing indicated a statistically significant difference. Kaplan-Meier estimates were calculated using GraphPad Prism Software (version 10.2.0). A p-value of 0.05 in the log-rank test indicated a statistically significant difference.

Supplementary Figure 1.

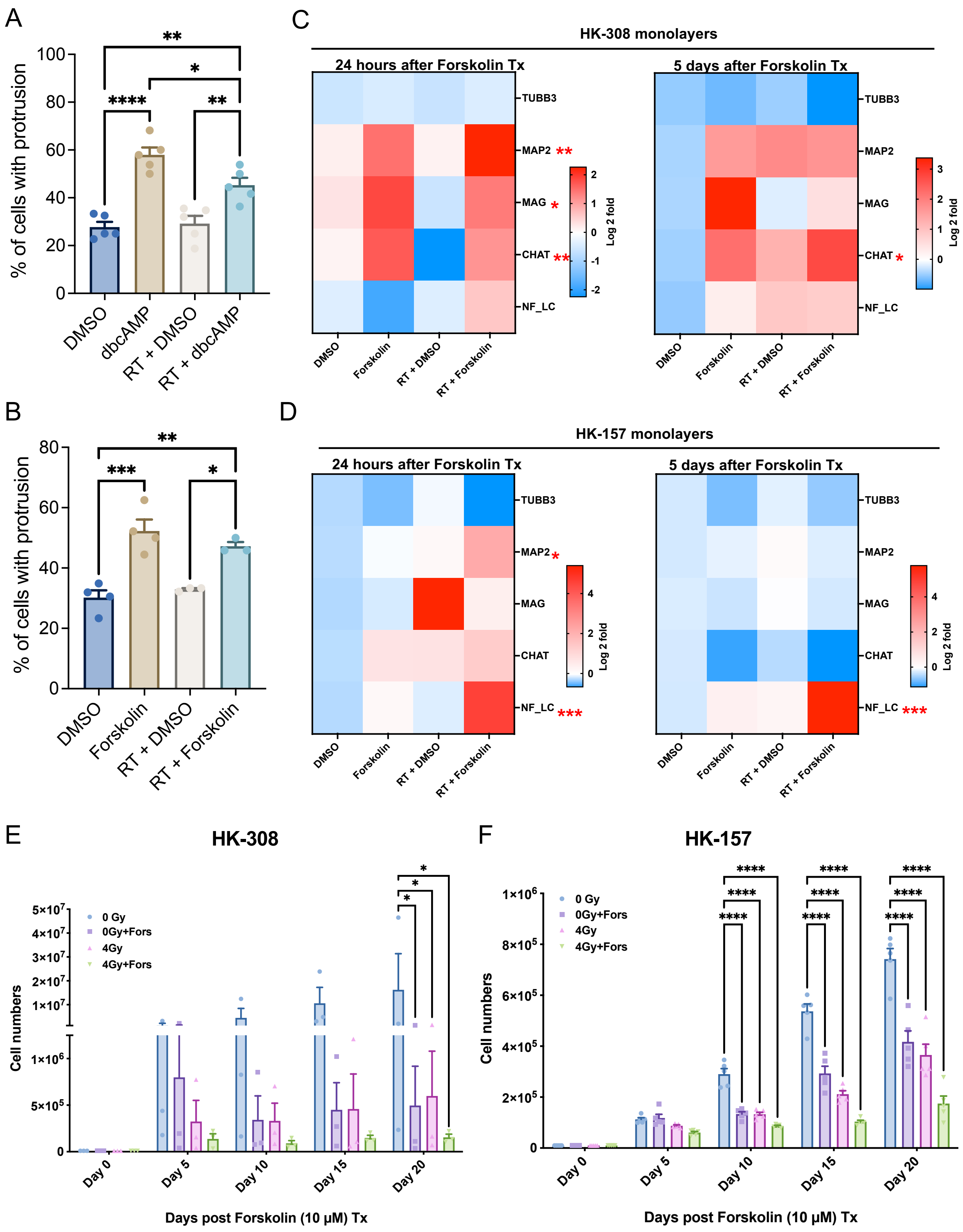

Supplementary Figure 1. Quantification of HK-374 cells exhibiting protrusions following dbcAMP or forskolin treatment, with or without radiation exposure **(A/B)**. Heatmaps showing the results of quantitative RT-PCR for the neuronal markers in both HK-308 **(C)** and HK-157 **(D)** monolayer cells treated with radiation (a single dose of 4 Gy) in the presence or absence of forskolin (10 μM) for 24 hours and 5 consecutive days. Treatment of both glioma cell lines HK-308 **(E)** and HK-157 **(F)** with radiation (a single dose of 4 Gy) and consecutive treatment of forskolin (10 μM) for 5, 10 15 or 20 days significantly inhibited cell proliferation. All experiments were conducted with at least 3 independent biological replicates (N=3-6). Statistical significance was determined using One-way ANOVA, and the values listed in the heatmaps were from the comparison of RT + Forskolin to RT + DMSO. * $p < 0.05$, ** $p < 0.01$, *** $p < 0.001$, **** $p < 0.0001$.

Supplementary Figure 2.

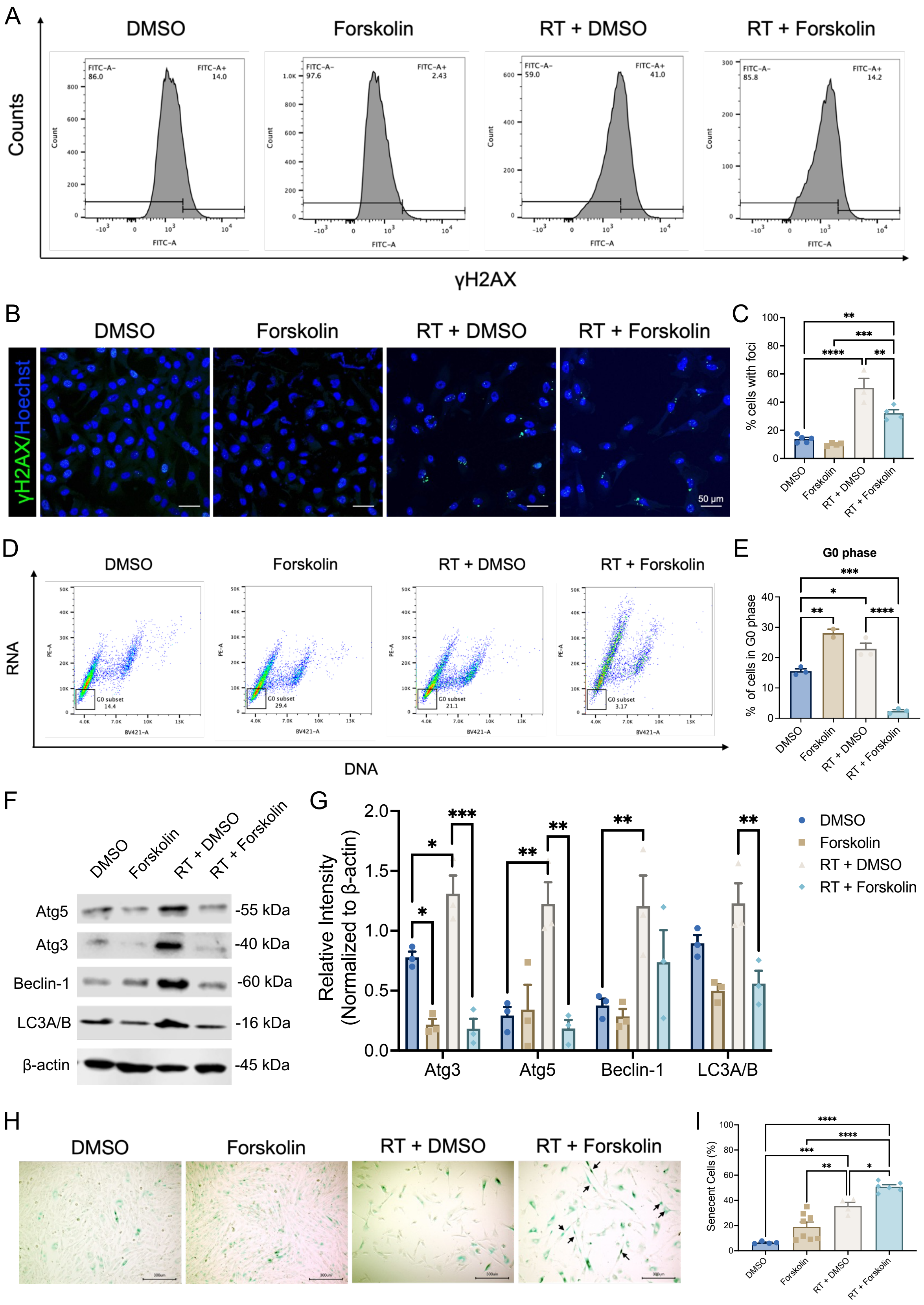

Supplementary Figure 2. FACS analysis **(A)** and immunofluorescent staining **(B)** for γH2AX in HK-374 cells treated with forskolin for 24 hours, along with quantification of cells exhibiting foci **(C)**. FACS analysis of the G_0 phase in HK-374 cells continuously treated with forskolin for 5 days. Dot plots depicting DNA and RNA content are shown in **(D)**, and the quantification of G0 phase cells is presented in **(E)**. Western blot analysis of autophagy markers in HK-374 cells continuously treated with forskolin for 5 days, with or without radiation **(F)**. Quantification of the western blot results using ImageJ **(G)**. β-galactosidase staining of HK-374 cells after continuous forskolin treatment for 5 days is illustrated in **(H)**, with corresponding quantification of the percentage of senescent cells in **(I)**. All experiments were conducted with at least 3 independent biological replicates (N=3-8). Statistical significance was determined using One-way ANOVA. * $p < 0.05$, ** $p < 0.01$, *** $p < 0.001$, **** $p < 0.0001$.

Supplementary Figure 3.

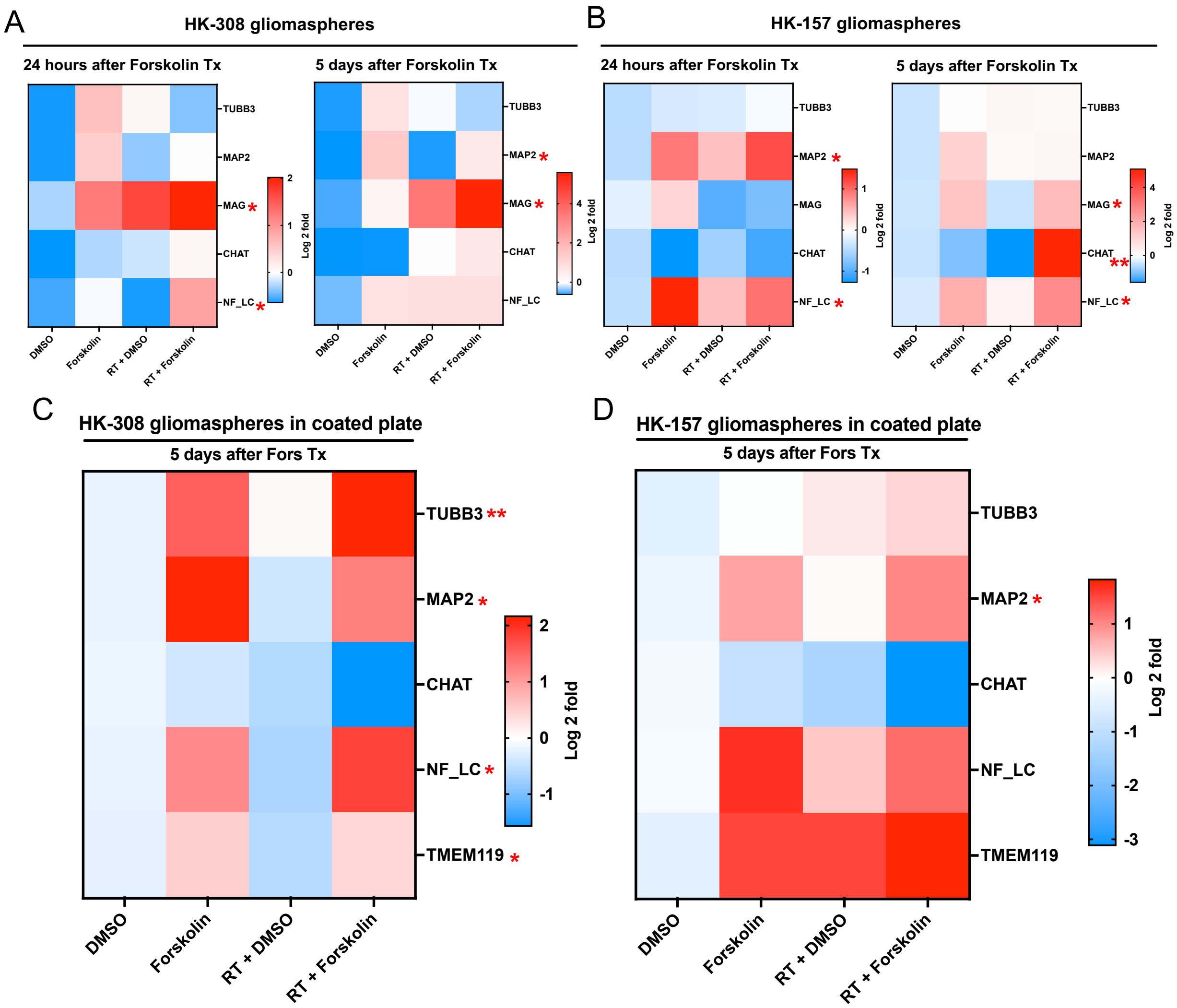

Supplementary Figure 3. Heatmaps showing the results of quantitative RT-PCR for the neuronal markers in both HK-308 **(A)** and HK-157 **(B)** glioma spheres treated with radiation (a single dose of 4 Gy) in the presence or absence of forskolin (10 μM) for 24 hours and 5 consecutive days in ultra-low adhesion plates, as well as in Poly-D-Lysine/Laminin coated plates after 5 consecutive forskolin treatment **(C/D)**. All experiments have been performed with at least 3 biological independent repeats (N=3-6). The *p*-values were calculated using One-way ANOVA, and the values listed in the heatmaps were from the comparison of RT + Forskolin to RT + DMSO. * *p*-value < 0.05, ** *p*-value < 0.01.

Supplementary Figure 4

A

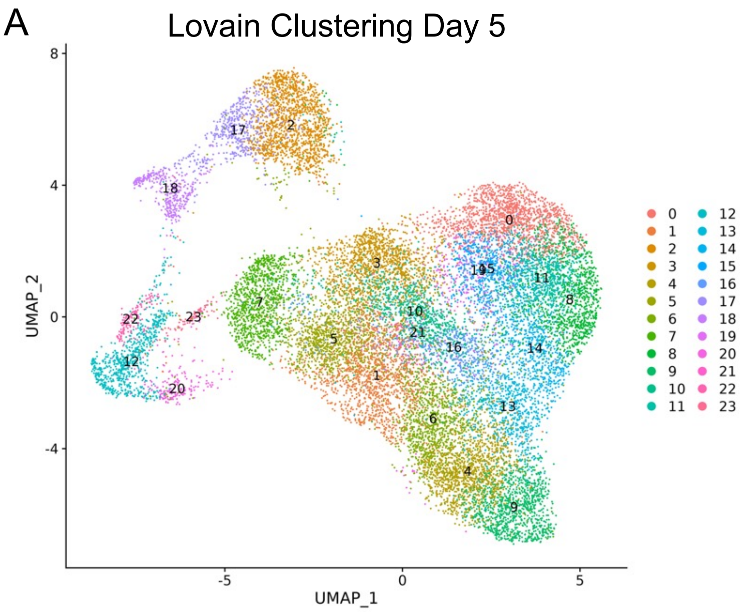

B

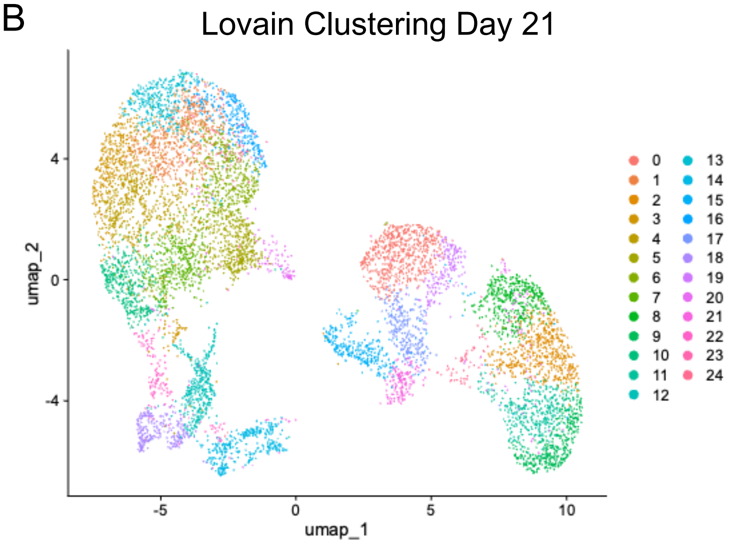

Supplementary Figure 4. UMAP plots of identified cell clusters from day 5 **(A)** and day 21 **(B)** samples.

Supplementary Figure 5

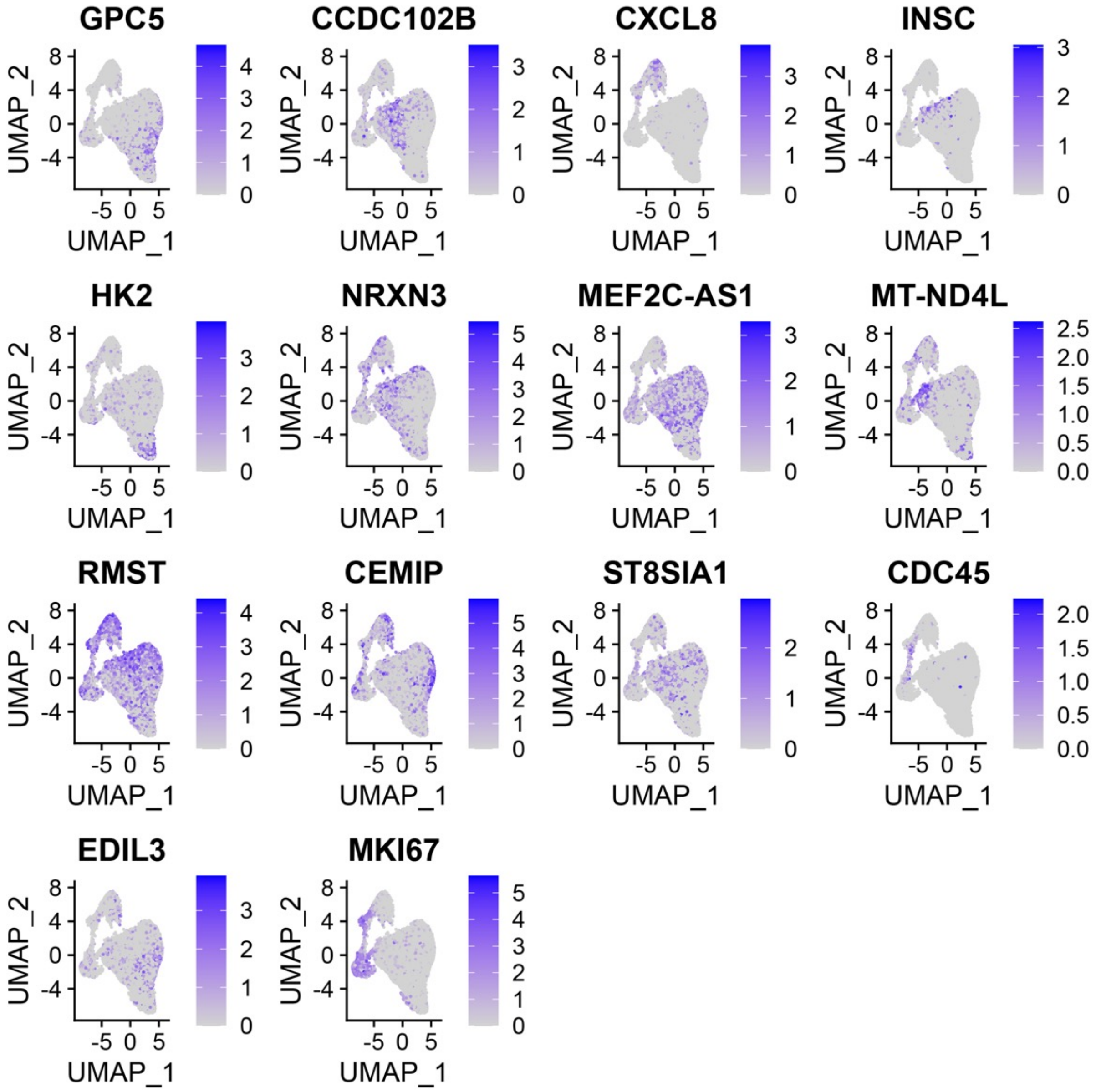

Supplementary Figure 5. UMAP plots showing marker genes on 5 days after forskolin treatment (7 days after irradiation).

Supplementary Figure 6

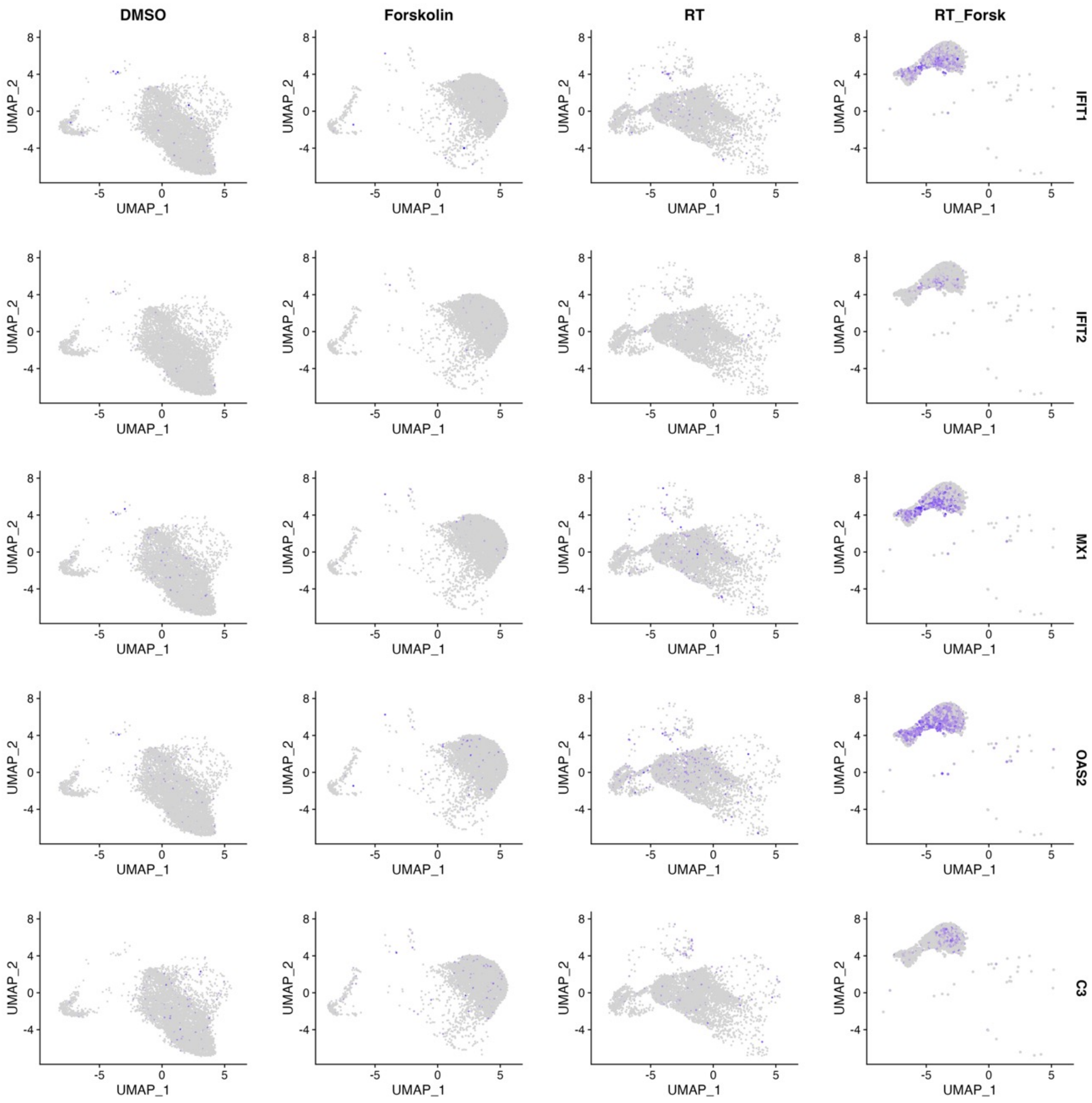

Supplementary Figure 6. Feature plots displaying the expression patterns of the top 5 Differentially Expressed Genes (DEGs) in microglia-like cells across all four experimental conditions.

Supplementary Tables

Supplementary Table 1

Species	Gene name	Primer sequence (5'–3')
Human	TUBB3	Forward: TTTGGACATCTCTTCAGGCC<br>Reverse: TTTCACACTCCTTCCGCAC
Human	MAP2	Forward: CTTCAGCTTGTCTCTAACCGAG<br>Reverse: CTGCAACTATTCAAGGAAGTGG
Human	MAG	Forward: CTACATTACCCAGACACGCAG<br>Reverse: TCTCGCTCTCGTACTTCTCTG
Human	CHAT	Forward: CATTGTGCAGCAGTTTGGGG<br>Reverse: TGCAAACCTCAGCTGGTCAT
Human	NF_LC	Forward: AGAGTGAAATGGCACGATACC<br>Reverse: ACTGGTTATGCTTCCCACG
Human	TMEM119	Forward: TGTCCACCCCAGTGTCTAA<br>Reverse: GTGTCAGGAAGCAGTCAGG
Human	PPIA	Forward: ATGCTGGACCCAACACAAAT<br>Reverse: TCTTTCACTTTGCCAAACACC

Supplementary Table 2. Patient demographics and TCGA-classification of GBM subtypes.

Line	Origin	Age	Sex	TCGA subtype	Culture P53 CN	EGFRvIII	PTEN	MGMT
HK-374	Primary GBM	45	M	classical	Loss "mosaic"	Positive	Positive	not methylated
HK-157	Primary GBM	54	F	proneural	wt	Negative	Positive	Unknown
HK-308	Recurrent GBM	50	F	mesenchymal	Unknown	Positive	Positive	not methylated

Supplementary Table 3. Cell counts and viability of the GBM cells cultured in the Poly-D-Lysine/Laminin coated plates

Experimental Group	Day 5		Day 21	
	Cell counts	Cell viability	Cell counts	Cell viability
DMSO_1	2.21 x 10^6/ml	95 %	2.47 x 10^6/ml	79 %
DMSO_2	3.03 x 10^6/ml	91 %	1.17 x10^6/ml	70 %
DMSO_3	2.24 x 10^6/ml	95 %	2.01 x10^6/ml	74 %
Forskolin_1	1.48 x 10^6/ml	89 %	1.72 x10^6/ml	67 %
Forskolin_2	1.66 x 10^6/ml	81 %	1.41 x10^6/ml	64 %
Forskolin_3	1.8 x 10^6/ml	91 %	1.40 x 10^6/ml	54 %
RT + DMSO_1	9.31 x 10^5/ml	83 %	1.77 x 10^6/ml	60 %
RT + DMSO_2	1.21 x 10^6/ml	84 %	1.72 x10^6/ml	62 %
RT + DMSO_3	1.22 x 10^6/ml	89 %	1.77 x 10^6/ml	67 %
RT + Forskolin_1	3.14 x 10^5/ml	70 %	9.40 x 10^5/ml	51 %
RT + Forskolin_2	2.88 x 10^5/ml	58 %	1.22 x 10^6/ml	56 %
RT + Forskolin_3	3.82 x 10^5/ml	68 %	1.11 x 10^6/ml	59 %

Supplementary Table 4. Percentage Composition (Day 5) for single cell sequencing

Condition	DMSO	Forskolin	RT	RT + Forskolin
Astrocyte-like	0.887393	18.07093	0.705972	0.301594
Dividing	3.151775	4.522424	5.8195	16.15683
G2.M	1.499388	0.206418	6.601794	0.08617
Glycolytic	33.35373	1.332333	1.335623	0.129255
Inhibitory Neuron-like	15.34578	1.632576	22.76283	0.215424
High MT count	0.0459	0.056296	20.56859	0.430849
Mesenchymal	4.023868	0.450366	5.456974	0
Microglia-like	0.0612	0.03753	0.381607	59.15554
NPC-like	15.28458	0.78814	30.24232	0.17234
Neuron-like	11.39841	28.33552	2.022515	22.66265
OPC-like	12.959	2.139238	3.415379	0.215424
RG-like	0.413097	4.691312	0.114482	0
Vascular-like	1.575887	37.73691	0.57241	0.473934

Supplementary Table 5. Percentage Composition (Day 21) for single cell sequencing

Condition	DMSO	Forskolin	RT	RT + Forskolin
Astrocyte-like	12.69	38.22	12.62	7.59
Dividing	19.27	5.94	15.98	8.56
G2.M	3.9	0.96	3.05	1.09
Glycolytic-like	1.11	0.03	0.31	0.04
Inhibitory Neuron-like	3.79	0.2	3	0.19
High MT count	7.57	1.73	7.12	14.71
Mesenchymal-like	25.84	11.38	26.31	2.41
Microglia-like	0.89	4.51	1.07	38.95
NPC-like	13.36	10.68	19.75	15.45
Neuron-like	10.58	25.28	9.87	10.23
Vascular-like	1	1.06	0.92	0.78

Supplementary Table 6. Confidence intervals for stem cell frequency (%)

Groups	Lower	Estimate	Upper
Corn oil	1.20772947	1.63934426	2.22222222
Forskolin	0.12487512	0.16207455	0.21052632
RT + Corn oil	0.15146925	0.19120459	0.24154589
RT + Forskolin	0.03066262	0.04061738	0.05379236

Supplementary Table 7. Pairwise tests for differences in stem cell frequencies

Group 1	Group 2	Chisq	DF	Pr(>Chisq)
RT + Forskolin	RT + Corn oil	96.6	1	8.37e-23
RT + Forskolin	Forskolin	73.3	1	1.1e-17
RT + Forskolin	Corn oil	313	1	5.7e-70
RT + Corn oil	Forskolin	1.44	1	0.23
RT + Corn oil	Corn oil	125	1	5.19e-29
Forskolin	Corn oil	142	1	7.84e-33

Reference:

1. E. Vlashi *et al.*, In vivo imaging, tracking, and targeting of cancer stem cells. *Journal of the National Cancer Institute* **101**, 350-359 (2009).
2. D. R. Laks *et al.*, Large-scale assessment of the gliomasphere model system. *Neuro Oncol* **18**, 1367-1378 (2016).
3. H. D. Hemmati *et al.*, Cancerous stem cells can arise from pediatric brain tumors. *Proc Natl Acad Sci U S A* **100**, 15178-15183 (2003).
4. K. Bhat *et al.*, The dopamine receptor antagonist trifluoperazine prevents phenotype conversion and improves survival in mouse models of glioblastoma. *Proc Natl Acad Sci U S A* **117**, 11085-11096 (2020).
5. Y. Hu, G. K. Smyth, ELDA: extreme limiting dilution analysis for comparing depleted and enriched populations in stem cell and other assays. *J Immunol Methods* **347**, 70-78 (2009).
6. S. X. Ge, E. W. Son, R. Yao, iDEP: an integrated web application for differential expression and pathway analysis of RNA-Seq data. *BMC Bioinformatics* **19**, 534 (2018).
7. A. Bhaduri *et al.*, Outer Radial Glia-like Cancer Stem Cells Contribute to Heterogeneity of Glioblastoma. *Cell Stem Cell* **26**, 48-63 e46 (2020).
8. V. Bergen, M. Lange, S. Peidli, F. A. Wolf, F. J. Theis, Generalizing RNA velocity to transient cell states through dynamical modeling. *Nat Biotechnol* **38**, 1408-1414 (2020).
9. S. Gao, Y. Dai, J. Rehman, A Bayesian inference transcription factor activity model for the analysis of single-cell transcriptomes. *Genome Res* **31**, 1296-1311 (2021).
